# Controlled Release of Madecassoside and Asiaticoside of *Centella asiatica* L. Origin from Sustainable Cold-Processed Topical Formulations

**DOI:** 10.3390/molecules29235583

**Published:** 2024-11-26

**Authors:** Monika Krzyżostan, Agata Wawrzyńczak, Izabela Nowak

**Affiliations:** 1 Faculty of Chemistry, Adam Mickiewicz University in Poznań, Uniwersytetu Poznańskiego 8, 61-614 Poznań, Poland; monika.krzyzostan@amu.edu.pl; 2 Dr Koziej Instytut Badań Kosmetyków, Czerniakowska 58, 00-717 Warsaw, Poland

**Keywords:** madecassoside, asiaticoside, *Centella asiatica* L., sustainable topical formulations, low-energy emulsification, controlled release, kinetic parameters

## Abstract

*Centella asiatica* L. extract is a promising natural agent for the treatment of atopic dermatitis. It significantly reduces inflammation due to its immunomodulatory properties, mainly attributed to the presence of pentacyclic triterpenes, namely madecassoside and asiaticoside. Their incorporation into sustainable cold-processed topical formulations, such as emollient-rich emulsions and cosmetic gel containing natural hydrophilic polymers, should inhibit inflammation in atopic skin. Therefore, the objective of this study is to investigate the controlled release of madecassoside and asiaticoside isolated from *Centella asiatica* L., loaded into topical formulations, namely emollient-rich O/W and W/O emulsions and cosmetic gel, which could support the treatment of atopic dermatitis. The carriers of active substances have been prepared with sustainable emulsifiers, active substances, and emollients obtained by green technologies from food industry wastes. Low-energy methods during the carrier emulsification process were applied to reduce carbon footprints and preserve the valuable properties of the raw materials used. The influence of the *Centella asiatica* L. extract on the physicochemical properties of the formulations was studied, showing a satisfactory degree of stability of the formulations obtained. Moreover, factors that may influence the mechanism and kinetics of the release of madecassoside and asiaticoside, such as the concentration of the active substance, the pH of the dissolution medium, and the type of the carrier, have been tested and widely discussed.

## 1. Introduction

*Centella asiatica* L. is a herbal plant from the Apiaceae family and Asiatica Linn. Species [1,2]. It is a perennial plant growing around water reservoirs throughout the majority of tropical and subtropical regions, such as India, Pakistan, Sri Lanka, Madagascar, South Africa, China, Korea, Japan, and Taiwan [3,4]. *Centella asiatica* L. has been widely used in Ayurvedic medicine for thousands of years to treat skin diseases, such as acne, leprosy, lupus, ulcers, eczema, psoriasis, scleroderma, vitiligo, burns, wounds, and scars [2,5]. Moreover, it has a long history of internal use as a treatment for nervous system illnesses, such as sleep disorders, depression, and Alzheimer’s disease, due to its calming effect, improving memory and concentration [6]. The main bioactive compounds of *Centella asiatica* L. responsible for the therapeutic effects of the plant are pentacyclic triterpenoids, namely asiaticoside and madecassoside, and their aglycones, such as asiatic acid and madecassic acid [4,5,7]. The content of the triterpene saponin fraction varies from 1 wt.% to 8 wt.%, depending on the plant’s growth location [5]. Madecassoside (asiaticoside A) represents ursane-type triterpene glycoside and is the most abundant triterpenoid of this plant, with an asiaticoside as a second one. Other substances isolated from the aerial part of *Centella asiatica* L. include monoterpenes, sesquiterpenes, tannins, phytosterols, flavonoids, essential oils, amino acids, and vitamins, such as vitamin C, vitamin B1, and vitamin B2 [4,5]. Pentacyclic triterpenoids offer a wide range of pharmacological properties related to the anti-inflammatory, immunomodulatory, antioxidant, antiviral, antibacterial, wound healing, neuroprotective, cardioprotective, hepatoprotective, anxiolytic, antifibrotic, anti-arthritic, anti-tumor, and anti-depressant activities [5,7]. Over the years, numerous studies have been conducted on the release of madecassoside and asiaticoside from intravenously or orally administered carriers, such as nanogels [8], alginate-chitosan nanoparticles [9,10], solid dispersion [11,12], capsules [13], and phospholipid-based naturosomes [14].

*Centella asiatica* L. extracts containing asiaticoside and madecassoside, due to the ability to stimulate collagen synthesis in human fibroblasts and lipolytic, anti-inflammatory, immunomodulatory, antibacterial, and antioxidant activities, are widely used in dermatology and cosmetology for treatment of acne, burns, wounds, psoriasis, vitiligo, and as a preventive agent against stretch marks and skin aging [4,6,7]. Moreover, *Centella asiatica* L. has been used in skin care products for restoration of skin firmness and elasticity, as well as in body care products to reduce cellulite and stretch marks [5,15]. Due to its numerous applications in the treatment of skin diseases, the possibility of using a standardized *Centella asiatica* L. extract in dealing with atopic dermatitis has also been explored. Characteristic symptoms of atopic dermatitis are erythematous and papulovesicular eruptions, which frequently develop into oozing, crusting, and persistent and severe itching, resulting from dry skin [16]. Madecassoside and asiaticoside, present in the standardized ethanolic extract of *Centella asiatica* L. or isolated from this plant as pure chemical compounds, act as a responsive factor to the key inflammatory processes occurring in the skin with atopic dermatitis. The immunomodulatory and anti-inflammatory properties of *Centella asiatica* L. extract and isolated triterpenoids were confirmed using in vitro and in vivo studies conducted by independent research groups [17,18,19]. 2,4-dinitrochlorobenzene (DNCB)-induced skin inflammation has been tested by Lee et al. by using in vitro and in vivo mouse models of atopic dermatitis skin [17]. In addition, the effect of the titrated extract of *Centella asiatica* L. (TECA) in a phthalic anhydride-induced atopic dermatitis animal model was studied by Park et al. [18]. Both groups confirmed that *Centella asiatica* L. extract inhibits hyperkeratosis and infiltration of inflammatory cells in atopic dermatitis lesions. These studies indicate that extracts of *Centella asiatica* L. or triterpene saponins isolated from this plant can be considered safe and effective therapeutic agents supporting atopic dermatitis treatment. Opposite to the glucocorticosteroids or calcineurin inhibitors commonly used during atopic dermatitis treatment, they do not show any side effects, such as skin atrophy or burning and stretch marks [19]. Therefore, studies on the release of triterpenoids from *Centella asiatica* L. from a variety of carriers applied to the skin have been recently conducted [20]. Among them, specific types of nanocarriers have been applied, namely nanoemulsions [21], solid lipid nanoparticles (SLN) [22], nanostructured lipid carriers (NLC) [23], polymeric nanoparticles [24], and liposomes [25]. Also, film-forming polymeric solutions (FFPS) [26,27], bilayer microneedles [28], ultra-fine cellulose acetate fiber mats [29], and electrospun double-layered nanocomposite membranes [30] were tested as topical carriers for the *Centella asiatica* L. extracts.

Due to the increasing consumers’ demands and obligations of the European Green Deal implementation, the recent trends in the cosmetic industry strongly move towards sustainability, which plays an important role in protecting ecosystems and biodiversity as it brings the possibility of obtaining environmentally friendly raw materials. This may be achieved by reducing carbon footprint and waste production, optimizing production processes, using green technologies, and applying ingredients obtained from agro-food wastes [31]. However, designing and marketing high-quality sustainable, eco-friendly, and biodegradable formulations stands as a big challenge to the cosmetic industry. There are several strategies for achieving sustainability in cosmetic products, with one based on replacing synthetic raw materials with those processed according to the green chemistry principles or COSMOS-approved natural ingredients [32]. Another strategy is based on lowering energy consumption during the acquisition of raw materials and production of cosmetics. This approach brings the reduction of the amount of greenhouse gases released, expressed as carbon dioxide equivalent units. As a result, studies regarding the development of sustainable cosmetic carriers using low-energy processes appear [33,34]. The study conducted by Tamburic et al. confirmed that using hot-cold emulsification, which is between the cold and hot manufacturing process of emulsions, saved approximately 82% (for O/W type emulsion) and 86% (for W/O type emulsion) of thermal energy in comparison to the hot process [33]. Moreover, Raposo et al. compared costs of the cold emulsification to the hot process and found that cold-processed O/W emulsions saved 67% in electrical, 36.7% in water, and 17.1% in the total production expenses [34]. The cold-processed emulsions allow not only to optimize production costs and minimize the carbon footprint but also enable the preservation of the valuable properties of natural ingredients, specifically oils used in the formulation, since oil-based ingredients under high temperature conditions are particularly susceptible to the oxidation process, resulting in the formation of free radicals [35,36]. The use of carriers based on oxidized oils can intensify inflammation on the skin, especially with atopic dermatitis [37]. Low-temperature emulsions processing, which supports the stability of lipid-based raw materials, becomes even more important when dealing with lipid-rich cosmetic formulations, so-called “emollient plus” products. The treatment of atopic dermatitis can also be supported by the dermal application of this type of product, as they minimize systemic effects and give good results in achieving remission of disease [38,39].

Conventional carriers such as W/O, O/W emulsions, and gels are the most commonly used types of formulations in the cosmetic industry. Therefore, studying the release of triterpenoids from *Centella asiatica* L. with high anti-inflammatory and immunomodulatory potential will provide important information on the potential of the most optimal type of carriers. However, there is very limited research available on the release of anti-inflammatory triterpenoids, such as madecassoside and asiaticoside, from sustainable, environmentally friendly, and cold-processed conventional topical formulations, which can support the treatment of atopic dermatitis. For this reason, we have developed three types of topical carriers obtained by a cold process with low energy consumption and a noteworthy reduction in carbon footprint. A variety of emollient-rich carriers, namely O/W and W/O emulsions, were designed, taking into account the needs of the atopic skin, as they contained 31 wt.% and 21 wt.% of emollients for W/O and O/W emulsion types, respectively. Special emphasis was paid on replacing the conventional raw materials with their substitutes obtained in accordance with the green chemistry principles or derived from the agro-food industry waste. The high potential of their biodegradability and low toxicity to aquatic organisms was taken into account, as well. All of the raw materials used to prepare the formulations were COSMOS-approved and met the requirements of Ecocert standards. The obtained topical formulations were evaluated towards their suitability for madecassoside and asiaticoside controlled release, with a special emphasis on the possibility of applying to skin suffering from atopic dermatitis. An important aspect of our research was also to verify the effect of pH on the release profile of madecassoside and asiaticoside from the tested carriers, which will have a direct influence on the therapeutic effectiveness of the topical formulations used, as one of the elements of pathophysiology of atopic dermatitis is an imbalance in the pH of the skin acidic mantle, which has been recognized as a regulating factor of skin barrier permeability and homeostasis of stratum corneum [40,41]. To the best of our knowledge, there is very little data available on the parameter optimization (e.g., pH of the medium, concentration of the active substance) and kinetic study for the controlled release from cold-processed topical formulations serving as carriers for triterpenoids of *Centella asiatica* L. origin with a potentially therapeutic effect in the atopic skin.

## 2. Results and Discussion

### 2.1. Characteristic of Cold-Processed Emulsions and Gel

#### 2.1.1. Sustainability of the Topical Formulations

During the development and preparation of carriers for saponin triterpenoids derived from *Centella asiatica* L., special emphasis was paid on replacing conventional raw materials with their substitutes obtained in accordance with the green chemistry principles, derived from the food industry by-products, and characterized by high values of biodegradability (at least 60% in 28 days, according to OECD methods). Moreover, low toxicity to aquatic organisms was also taken into account. It was not only to follow the requirements of the modern cosmetic market but also to take into account the potential applications of the obtained products as cosmetics for atopic skin. Emulsions and gel were obtained with COSMOS-approved raw materials, meeting the requirements of Ecocert standards. Therefore, the obtained topical formulations can be recognized as sustainable [42]. They contain vegan ingredients of natural origin that were obtained by physical, biotechnological, or chemical methods in accordance with the principles of green chemistry. At least 10% of all raw materials used in each formulation come from upcycling of agro-food by-products. For example, Tocomix L70-IP Organic CO contains tocopherols with antioxidant properties derived from by-products in the refinery process of soybean oil, whereas polymeric emollient Citropol H was obtained from side-stream turpentine generated during the paper-making process. Moreover, E-Leen green C is a vegetal-based preservative that contains 100% renewable carbon. It shows two-in-one action, namely, it protects formulations against microbiological contamination, especially from bacteria and yeasts, and it has a beneficial effect on the skin epidermal barrier thanks to its moisturizing and re-fatting properties. Besides pentylene glycol, it also contains glyceryl caprylate/caprate, serving as a humectant and a non-ionic co-emulsifying emollient that is mild to the skin. On the other hand, the hemp oil used in the manufacturing of Galehemp^®^ OW is certified as a non-psychoactive ingredient according to European regulations, as it comes from the European type of hemp, which is a non-psychoactive plant (THC less than 0.2 wt.%). The details regarding the origin of the raw materials, their production methods, biodegradability, and toxicity to aquatic organisms are presented in Table 1.

When designing and processing sustainable carriers of active substances, both in the cosmetic and pharmaceutical industries, low energy consumption is essential for reducing the carbon footprint in the environment and should become a standard. For this reason, we have developed three types of sustainable topical carriers for madecassoside and asiaticoside, namely O/W and W/O emulsions and gel, which were obtained by cold processes. Cold-processed emulsions are more difficult to stabilize due to the lack of possibility of using solid wax thickeners in the oil phase. Therefore, other solutions are required to obtain a stable product. A thorough screening of the raw materials market allowed us to select and test modern non-ionic emulsifiers, which enabled us to perform the cold emulsification process. The non-ionic EWO emulsifier was selected for obtaining W/O emulsion because of its natural origin and ability to stabilize such systems. Moreover, it gives a long-lasting moisturizing effect, and it is characterized by good compatibility with sensitive skin, which is especially important in cosmetic products dedicated to people suffering from atopic dermatitis. On the other hand, the non-ionic emulsifier Galehemp OW was applied during O/W emulsion synthesis. Apart from its emulsifying properties, it provides emollient and moisturizing properties. As one of the few O/W emulsifiers, it is suitable for preparing cold-processed emulsions. Cold-processed emulsification with non-ionic emulsifiers did not require the thermal energy normally used to heat the phases of emulsions and only mechanical energy for the dispersion of droplets and their homogenization was necessary. As a result, energy consumption and economic costs for obtaining emulsion-type carriers for testing controlled release of madecassoside and asiaticoside were significantly reduced. In the case of preparing gel-type carriers highly efficient in the cold process, natural polymers, such as xanthan gum, alginate, and tapioca starch, were used.

By paying special attention to the environmental aspects of designing and formulation of topical carriers, we were also able to respond to the global trends and the European Green Deal principles, which assume that the EU economy will become zero-emission by 2050 [43]. In addition, the implementation of low-energy processing during emulsion synthesis is cost-efficient because it allows us to reduce the emulsification temperature by more than 50 °C when compared to the conventional processes commonly used in the cosmetic and pharmaceutical industries. According to Raposo et al., cold emulsification gives a reduction of 67% in electrical, 36.7% in water, and 17.1% in total production expenses when compared to the hot process [34].

#### 2.1.2. Organoleptic Assessment and Physicochemical Properties of Topical Formulations

The initial physicochemical parameters, such as pH, viscosity, and density, in the formulations prepared were measured. The results are given in Table 2.

All of the synthesized topical emulsions were homogeneous, characterized by a light color and a delicate odor, typical of the raw materials used. The gel, on the other hand, was a homogeneous, semi-transparent, and opalescence product without any odor (Figure S1). The appearance of the initial formulations did not differ from that of the formulations after the introduction of 10 wt.% of *Centella asiatica* L. extract. Also, the density of all products was similar and oscillated around the value of 1 g/mL.

As can be seen in Table 2, the pH of the obtained formulations ranges from 5.45 and 5.32 for the O/W and W/O emulsions, respectively, to a value of 4.93 for the gel. These differences are mainly due to the ingredients used to obtain the products. The introduction of the extract results in a slight decrease in the pH value of all formulations. The lower pH of the gel is most likely due to the Instathix^®^ thickener used, which is based on a lactic acid derivative. As shown, topical formulations of an acidic nature can lower the pH of the skin without destroying its protective barrier [44]. Additionally, we may assume that the higher acidity of the gel-based formulation can have a positive effect on the skin affected by atopy, where pH is usually elevated. This was demonstrated in a recent study by Andrew et al., where the use of a formulation with pH = 4.0 helped to maintain the acidic pH of the skin surface and supported the delivery of physiologic lipids, giving a reduction in sensitivity to irritants and allergens, which is typical to atopic dermatitis [45]. Nevertheless, the pH of all formulations obtained is within the range considered to be the optimal pH of the human skin, i.e., 4.1–5.8 [46].

Due to their physicochemical form and the raw materials used, the resulting formulations differed markedly in viscosity, reaching 605.40 cP for the O/W emulsion and 6548.20 cP for the W/O emulsion (Table 2). Moreover, these values decreased noticeably when 10 wt.% of *Centella asiatica* L. extract was added to the formulation, yielding 394.20 cP and 4993.60 cP for the O/W and W/O emulsion, respectively. Such high viscosity values for W/O emulsions are mainly due to the fact that the formulations rich in oils and lipids naturally generate higher viscosity products. In addition, most commercially available viscosity modifiers are soluble in the aqueous phase, and their performance in the W/O emulsions is limited. Viscosity-modifying agents influence the microscopic structure of the formulation, like droplet size distribution, which, in turn, impacts the macroscopic behavior of the product. Many other parameters can also affect the final viscosity of the resulting products, including the speed of the rotation and temperature during homogenization [47].

#### 2.1.3. Stability Tests of O/W and W/O Emulsions and Gel with *Centella asiatica* L. Extract

**Figure 1:**
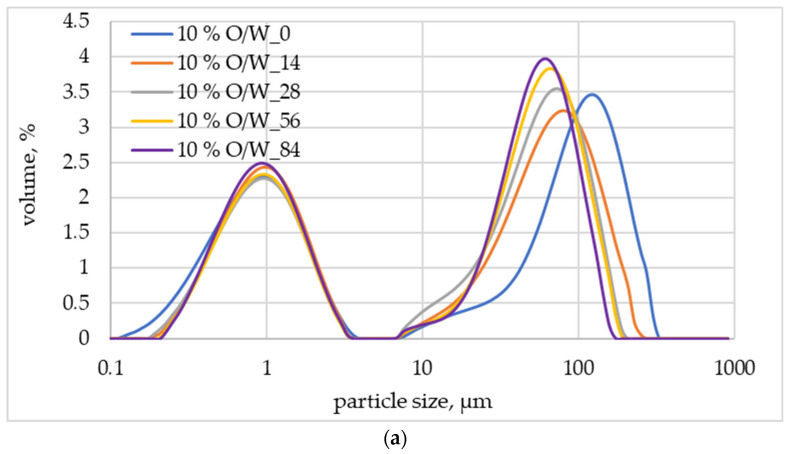

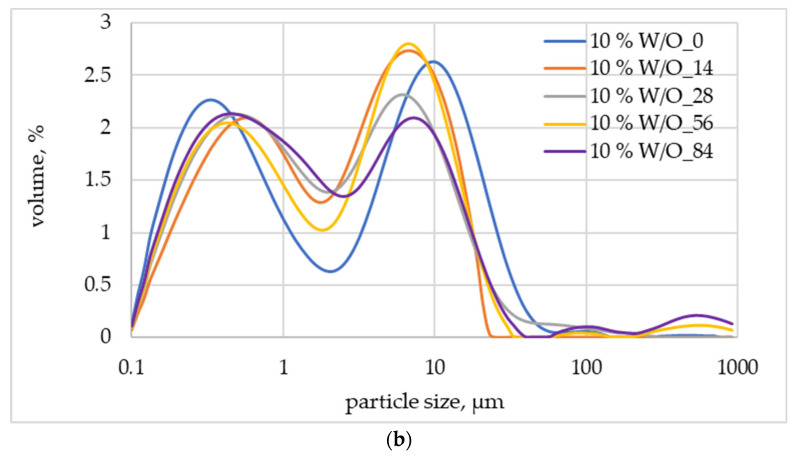
Particles (droplets) size distributions of O/W (**a**) and W/O (**b**) emulsions containing 10 wt.% of *Centella asiatica* L. extract; the digit at the end of the symbol indicates the number of days the sample was stored at elevated temperature (45 °C).

The accelerated stability testing gives some predictions of long-term product stability under ambient conditions in shorter time periods. Storage of topical formulations at conditions exceeding ambient temperature can result in significant changes in their physical characteristics, namely particle size, rheology, and product structure. These changes significantly influence the stability of the product and are, among others, due to fluctuations in the components’ solubility, such as rheology-modifying agents or variations in the size of particulates and emulsion droplets [48]. Consequently, one of the parameters determining the stability of cosmetic emulsions is the uniform dispersion of their internal phase. An image of the emulsion particles (droplets) size distribution (PSD) can be obtained by the measurements based on the laser diffraction. The results of such measurements carried out for emulsions containing 10 wt.% of *Centella asiatica* L. extract are shown in Figure 1.

For all O/W emulsion-based samples, a clearly bimodal PSD of the dispersed phase could be observed (Figure 1a) with a first maximum around 1 µm and the second one ranging from 76.0 µm (t = 84 days) to 144.5 µm (t = 0). Moreover, no drastic changes in the PSD can be detected when the emulsion is stored at elevated temperatures. It can be seen that the smaller droplets of the internal phase were stabilized very well by the emulsifiers used. More distinct changes can only be noticed in the PSD of larger droplets and for measurements taken immediately after preparation of emulsion (t = 0) and after 14 days of its storage at 45 °C, resulting in the peak maximum shift from 144.5 µm to 91.0 µm. After the emulsion was stabilized by the emulsifiers used, subsequent measurements showed a stable droplet size distribution of the dispersed phase. The results show that stable products were obtained in which the formulation components, as well as the emulsification and homogenization conditions, were properly selected. As Kim et al. have shown, a suitable selection of these elements is vital for the stability of O/W emulsions, since a high shear mixing rate in emulsification may induce Brownian flocculation, which, in turn, accelerates the coalescence of tiny droplets, leading to a decrease in the long-term stability of the cosmetic O/W emulsion [49].

In contrast, a wider particle size distribution (0.1–60.0 µm) was obtained for the W/O emulsion-based product (Figure 1b), with two marked maxima at about 0.6 µm and 8.0 µm. A more pronounced difference can be observed between the freshly obtained emulsion and the emulsion stored for 14 days at elevated temperature, for which the peak maxima shift from 0.35 µm and 10.0 µm (t = 0) to 0.50 µm and 6.5 µm (t = 14 days). Moreover, for samples stored for 56 days and 84 days, a minor tendency toward agglomeration of the inner phase droplets can be assumed, as manifested by the appearance of small peaks at values of about 100 µm and 550 µm. Such an unfavorable increase in the particle size of the dispersed phase, which can affect the stability of the product, is caused not only by an increase in the absolute size of individual droplets (coalescence), but it may also involve the formation of aggregated structures of the dispersed phase, which consequently increase effective droplet size. This phenomenon, called flocculation, can be induced by many factors and is difficult to predict, although it is very often triggered by large differences in the density of the emulsion’s two phases [48]. Given this, a slightly higher tendency in W/O emulsion to destabilize over time may be anticipated when compared with its O/W counterpart.

Another parameter to assess the stability of the emulsion is zeta potential (ZP), which determines the repulsive forces of particles in the colloidal systems. Zeta potential analysis is performed based on ELS phenomena and measures electrophoretic mobility, which is the speed of movement of charged particles suspended in a liquid under the influence of an electric field. Then the electrophoretic mobility is converted into a zeta potential value, which can be used as a measure of the colloidal system’s stability [50]. Suspension samples with ZP greater than ±30 mV may be considered moderately stable, whereas ZP value close to zero suggest instability resulting from flocculation or coagulation processes. In general, the higher the zeta potential, the more probable the preservation of dispersion stability [51]. In the course of our research, the zeta potential was measured for samples of cosmetic emulsions and gel containing *Centella asiatica* L. extract immediately after their preparation and during their storage for 84 days under elevated temperature conditions (45 °C). Thus, the long-term stability of the obtained sustainable cold-processed topical formulations containing madecassoside and asiaticoside of *Centella asiatica* L. origin could be evaluated.

**Figure 2:**
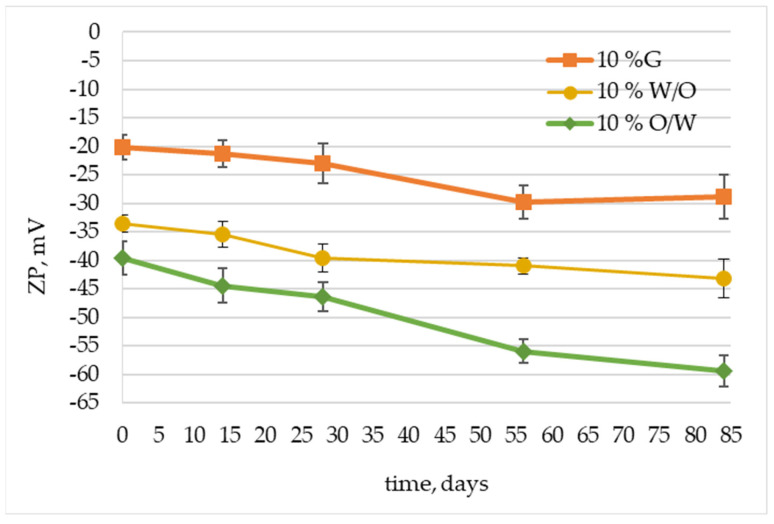
Zeta potential values measured in samples of oil-in-water (O/W) and water-in-oil (W/O) emulsions and gel (G) containing 10 wt.% of *Centella asiatica* L. extract.

As follows from the data in Figure 2, the emulsion-based samples immediately after preparation were characterized by satisfactory stability, as reflected by ZP values of −33.5 mV and −36.6 mV for W/O and O/W samples, respectively. Moreover, these values (expressed as modulus) increased over time and after 84 days reached −43.2 mV and −59.4 mV for W/O and O/W samples, respectively. Higher ZP values indicate stronger electrostatic interactions, so particles of the dispersed phase of the colloidal system undergoing such interactions will be strongly separated from each other, and this will result in higher dispersion stability. The highest stability, expressed as ZP value, was obtained for O/W cold-processed emulsion, which indicates the proper choice of emulsifier. This is worth noting, as the stabilization of this type of system is challenging without the classic emulsifiers normally used in the hot-processed emulsions. Thus, it can be concluded that the synthesized sustainable cold-processed W/O and O/W emulsions are long-term stable systems and can be used as carriers for active substances such as madecassoside and asiaticoside.

The gel sample had slightly lower ZP values ranging between −21.3 mV and −28.9 mV for the initial sample and the sample stored at 45 °C for 84 days, respectively (Figure 2). However, in the case of gels, such ZP values may also indicate a satisfactory level of stability. Gels constitute a specific example of colloids, as they are formed when there are so many particles of the dispersed phase present that they form a spatial network. Additional stabilization of the dispersed phase particles can be achieved by the chemical crosslinking reaction occurring in the case of gels formed by polymers. It is the case of the studied gel samples, as for the preparation of which natural polymers of plant origin, namely xanthan gum, alginate, and tapioca starch, were used. Therefore, gel-based formulations usually present a lower ZP value, as they are stabilized rather sterically than by electrostatic repulsions [52]. As such, despite ZP values that differ from those indicated in the literature for stable suspensions [51], the tested sustainable gel-based formulation can be used as a relatively stable carrier for active substances.

As mentioned before, physical changes observed at accelerated testing at elevated temperatures can be beneficial in establishing the stability of a product. However, quite often the instability of a product is initially not visible to the human eye, which can result in a false positive assessment of its long-term stability. In this situation, one of the solutions is to use the measurement based on static light scattering, where the backward reflected light results from multiple scattering of the photons on the suspension particles (droplets). It enables the quantification of light backscattering and transmission values, as well as the prediction of the long-term stability of suspensions without any manipulation, dilution, or disruption of samples [53]. Based on the results of light backscattering and transmission measurements, calculation of the TSI (Turbiscan stability index) parameter can be performed. TSI summarizes all the fluctuations of backscattering and transmission values in the sample and enables the evaluation of sedimentation, creaming, or flocculation occurrence. The changes in the TSI parameter in subsequent measurements indicate ongoing destabilization of the cosmetic sample. Consequently, the sample is stable when TSI tends toward zero and unstable when it reaches higher values [54].

**Figure 3:**
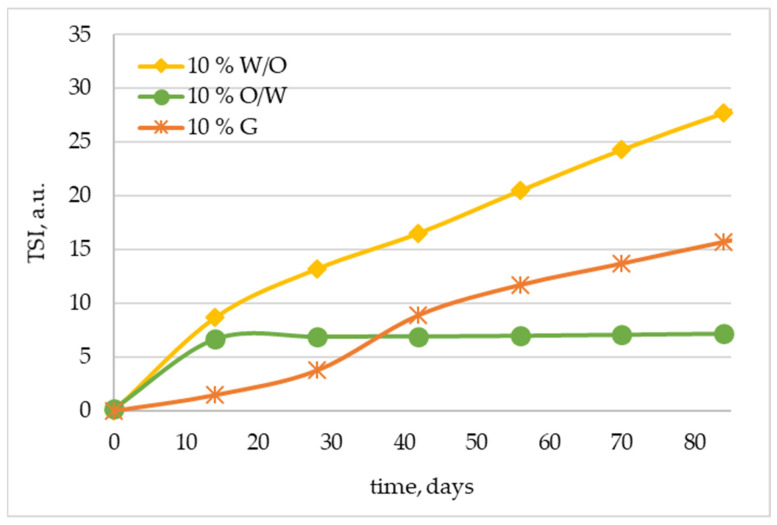
Stability of oil-in-water (O/W) and water-in-oil (W/O) emulsions and gel (G) containing 10 wt.% of *Centella asiatica* L. extract expressed as TSI values vs. time.

Figure 3 shows the results of our tests of the static light scattering for O/W, W/O emulsions, and gel samples containing 10 wt.% of *Centella asiatica* L. extract and stored for 84 days at elevated temperature (45 °C). The presented curves indicate some differences in the stability of the topical formulations obtained. The O/W emulsion-based sample appears as the most stable system, as the TSI parameter does not change throughout the accelerated stability test. At the initial stage of the study, the gel-based sample was also characterized by low TSI values, but after 30 days of storage, its stability deteriorated, as manifested by an increase in TSI values from 3.8 to 8.9 and a progressive increase in the value of this parameter up to TSI = 15.7 after 84 days. This is probably due to changes in the charge of the thickening agent used, which makes the repulsion forces in the gel matrix smaller, and therefore, the aggregation of particles may occur. Similar observations were also made by Ziółkowska et al. in the formulations based on carrageenan and poly(diallyldimethylammonium chloride) [55]. Our assumption can also be confirmed by the results of zeta potential measurements (Figure 2), where a change in the plot after 30 days of the sample’s storage is visible. Nevertheless, a certain degree of stabilization may still be provided by the dense packing of the gel network stabilized by the organic polymers present in the thickener used [52]. W/O emulsion appears to be the least stable system, as manifested by the steadily increasing value of the TSI parameter, which reaches 27.7 after 84 days of the accelerated stability test. This reduced stability was already indicated by measurements of the particle size distribution (Figure 1b), where additional peaks appeared in the range of higher values of PSD, indicating the beginning of the flocculation for samples stored for 56 and 84 days at elevated temperature. The larger the droplet size, the higher the velocity of creaming or sedimentation processes exhibited by increased TSI values. All these phenomena are disadvantageous to the emulsion’s long-term stability.

### 2.2. Controlled Release of Madecassoside and Asiaticoside from Sustainable Cold-Processed Topical Formulations

#### 2.2.1. HPLC Method Validation

To determine the amount of triterpenoids, i.e., madecassoside and asiaticoside, released from the various topical formulations, the HPLC technique was applied. The developed HPLC method was subjected to some validation tests to ensure the reliability of the analysis. As a first step, peaks from substances that can leach from the various cosmetic carriers were checked for potential overlapping with peaks from the analyzed active substances. Chromatograms obtained for formulations containing no active substances did not show the presence of peaks with retention times similar to that characteristic of the analyzed triterpenoids. Additionally, good separation of the peaks originating from madecassoside and asiaticoside was achieved, reaching 7.3 min and 11.5 min, respectively. Therefore, a simultaneous determination of both triterpenoids was feasible.

Table 3 shows the linear equations for calibration curves, their R^2^ parameters, LOD and LOQ values, as well as precision and accuracy, which were expressed as a percentage of recovery. Calibration curves for madecassoside and asiaticoside quantification are shown in Figure S2.

The developed HPLC method for simultaneous analysis of madecassoside and asiaticoside showed good linearity, with R^2^ values reaching 0.999. LOD and LOQ were 0.0045 mg/mL and 0.0136 mg/mL for madecassoside and 0.0053 mg/mL and 0.0159 mg/mL for asiaticoside, respectively. Moreover, the HPLC method used can be considered precise and accurate, as RSD was 0.87% for madecassoside and 1.29% for asiaticoside analysis, whereas the accuracy was close to 100% with the percent recoveries of madecassoside and asiaticoside as high as 104.71 ± 1.35 and 111.95 ± 0.98, respectively.

#### 2.2.2. Controlled Release Profiles

**Figure 4:**
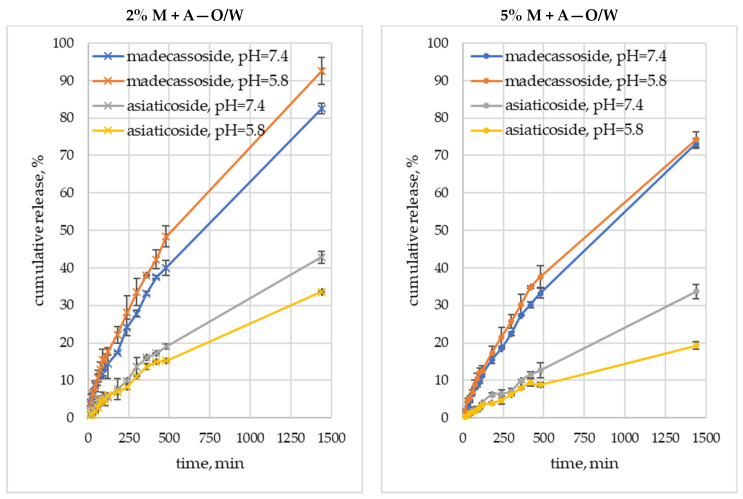

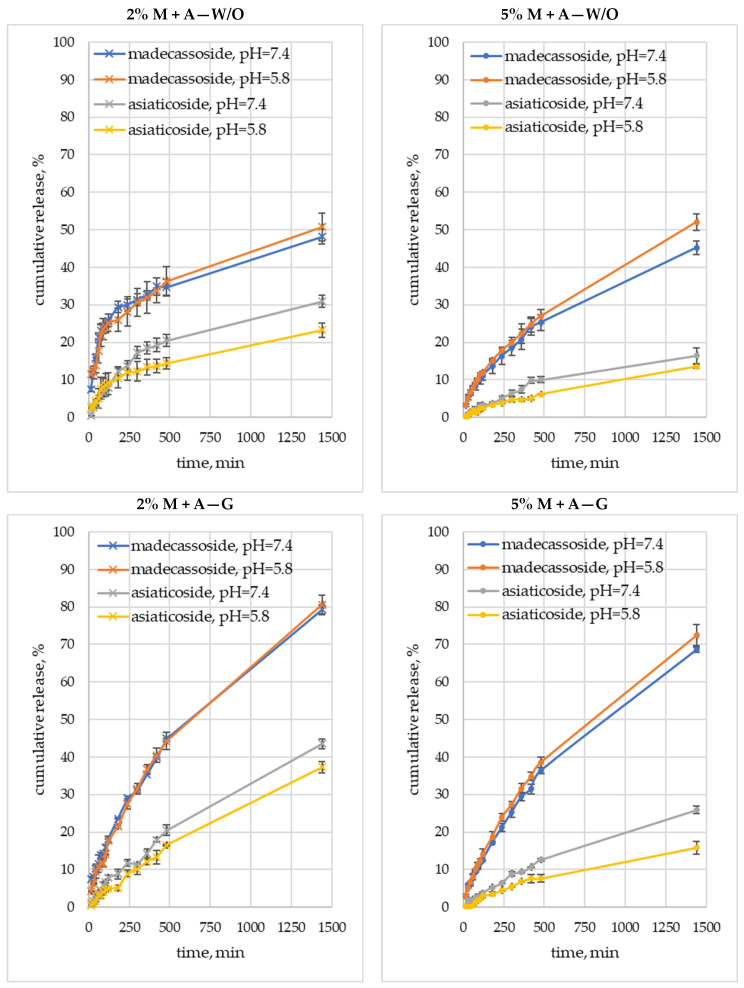
Controlled release profiles of madecassoside (M) and asiaticoside (A) from sustainable topical formulations in different acceptor fluids; O/W, W/O, and G abbreviations stand for oil-in-water or water-in-oil emulsions and gel, respectively.

The foundation of the dissolution test was set at the end of the 19th century by Noyes and Whitney and applied to the diffusion-controlled dissolution process. However, up to this date, several other models of dissolution have been proposed [56]. As such, testing the release of triterpenoids from *Centella asiatica* L. can be a valuable source of information on the selection of the best type of carrier, the concentration of the active substance, and pH values, at which madecassoside and asiaticoside obtain optimal release profiles. During our research, a methodology based on spikes with active substance standards was used. The prepared cosmetic formulations were enriched with 2 wt.% and 5 wt.% of madecassoside and asiaticoside and subjected to dissolution tests in two different buffer solutions at pH = 5.8 and pH = 7.4. Interestingly, as can be seen from the graphs in Figure 4, the introduction of larger amounts of active substances does not increase their cumulative release from the cosmetic matrix. Giuliano et al. also observed a similar correlation in the release profiles of rutin from Poloxamer 407-based hydrogels, which was not influenced by the increasing amount of active ingredient introduced into the cosmetic matrix [57]. Soe et al. indicate in their study that this might be explained in terms of the viscosity and the particle size of the carrier [58].

Nevertheless, the influence of the formulation type on the release profile is evident (Figure 4), indicating the O/W emulsion as the best carrier for the controlled release of triterpenoids tested. The highest amounts of madecassoside and asiaticoside were released from this system (≈90% and ≈40%, respectively) during 1440 min (24 h) of the test run. The second carrier that allowed the release of significant amounts of active substances was a gel, from which ≈80% and ≈40% of madecassoside and asiaticoside, respectively, were finally released. The least favorable environment for the release of triterpenoids found in *Centella asiatica* L. extract is created by the W/O emulsion. This is most likely due to the high viscosity of this matrix, as indicated by the data in Table 2. Increased viscosity of the matrix inhibits migration of the active substance to the boundary layer; hence, diffusion through the carrier may become the main parameter controlling the release processes. A similar relationship was observed by Dymek et al. when they studied the release of astaxanthin from different types of emulsions in topical application [59]. When comparing the release profiles of active substances from different emulsions, it is clear that the release of madecassoside, which has good solubility in hydrophilic systems, is favored by the more hydrophilic nature of the carrier. On the other hand, for W/O emulsions, in which the outer phase is lipophilic in nature, the difference between released madecassoside and asiaticoside is the smallest, which is due to the better solubility of asiaticoside in lipophilic systems, resulting in its easier access to the acceptor medium.

Since the physiology of atopic skin is strongly disturbed by the inflammation processes, it is commonly characterized by deviations in pH values [40,41]. Thus, when studying the release of active substances, the effect of the acceptor medium pH on the release profile should be considered [8].

In our study, the pH values of the acceptor solutions were selected in relation to the pH values of skin affected by atopic dermatitis (pH = 7.4) and healthy skin (pH = 5.8). Based on the correlations depicted in Figure 4, it can be seen that the influence of the acceptor medium pH is particularly marked in the case of the release of asiaticoside, making it possible to obtain higher release values of this substance at pH = 7.4. This may be advantageous in the care of atopic skin, especially at the beginning of the treatment, when the pH is still elevated due to the inflammatory processes taking place in the skin. With this course of asiaticoside release, it will be possible to apply its significant amounts, which will promote the leveling of inflammation and lowering of skin pH. Furthermore, the topical formulations we have prepared are characterized by a pH close to 5 (Table 2), which will naturally lower the pH of atopic skin during the treatment and promote the restoration of the skin’s natural acidic barrier. As can be seen from the release profiles shown in Figure 4, lowering the pH will slightly reduce the amount of asiaticoside released but will still promote the release of the second active ingredient, namely madecassoside. Our study not only gives information on the degree of released madecassoside and asiaticoside, but at the same time indicates good stability of these substances in the tested pH ranges. It is in good agreement with the results obtained by Puttarak et al., who also found that madecassoside and asiaticoside are rather stable in acidic and neutral pH and decompose under basic conditions with pH as high as 8.2 [60]. Thus, it can be considered that the topical formulations containing *Centella asiatica* L. extract obtained in the course of our study will enable comprehensive care of skin affected by atopy and with disturbed pH.

The trends described above were also confirmed by the data in Table 4, Table 5 and Table 6, which include the t_20%_ and X_480min_ parameters, indicating, respectively, the time at which the cumulative release value reached 20% and the release percentage achieved after 480 min of the dissolution test. It shows that the fastest 20% release of madecassoside was achieved for the W/O emulsion. In contrast, 480 min (8 h) after application of the formulation to the skin, the highest release from the O/W formulation can be expected, namely 48.3% and 15.2% for madecassoside and asiaticoside, respectively. The release values we achieved, especially those after 480 min (8 h), which is the time the formulation is usually kept on the skin during the day, should be considered satisfactory, given the data available in the literature. For example, Suksaeree et al. revealed low content of madecassoside and asiaticoside released from film-forming polymeric solutions (FFPS) using a Franz-type diffusion cell. The maximum release of madecassoside and asiaticoside after 8 h of testing was 13.4 and 14.8%, respectively [26]. In vitro experiments performed by Soe et al. demonstrated that the release of asiaticoside from in situ-created gel formulations through a semi-permeable membrane in the modified Franz diffusion cell does not exceed 10% after 8 h of testing [58]. On the other hand, in vitro release of asiaticoside from nanostructured lipid carriers containing dried (NLC-D) and glycolic (NLC-G) *Centella asiatica* L. extracts showed that after 8 h the release from NLC-D was approximately 20%, while release from NLC-G was 2-times higher during the same period of time [23]. In addition, a study by Suwantong et al. demonstrates that after 8 h of dissolution tests at 37 °C with electrospun cellulose acetate fiber mats containing asiaticoside, it is possible to achieve about 25% of asiaticoside cumulative release [29]. In a study conducted by Chowdahalli et al., the release of asiaticoside from solid lipid nanoparticles (SLN) incorporated in hydrogel was investigated by using the dialysis bag method. The release of asiaticoside in a sustained manner during 8 h was confirmed, and the amount of released substance was 88% [22]. High values of asiaticoside cumulative release after 8 h (approximately 50% to 60%) were also shown by Wichayapreechar et al. They used the dialysis method in phosphate buffered saline and applied it to niosomes and hyaluronic acid-modified niosomes loaded with *Centella asiatica* L. extract [61]. Contrary, Suhail et al. performed in vitro assessment of chitosan-based polymeric pH-responsive nanogels for sustained delivery of madecassoside, obtaining approximately 60% of cumulative release after 8 h of testing at pH = 7.4 [8]. Moreover, Lu et al. conducted an in vitro study of madecassoside release from nanoemulsions and showed that around 65% of madecassoside was released after 8 h [21].

#### 2.2.3. Kinetics of Madecassoside and Asiaticoside Release

The quantitative interpretation of the results obtained during release tests can be supported with mathematical equations and kinetic models in which the released amount of the substance tested is expressed as a function of the test time. Zero and first-order analysis, as well as Higuchi, Hixson–Crowell, and Korsmeyer–Peppas models, are usually used as analytical definitions of the Q(t) function. To assess the fitting of a model equation to the experimental data, the regression coefficient R^2^ is commonly used with R^2^ values close to 1, indicating the most suitable model of kinetics [62].

In the course of our study, four different kinetic models were evaluated for their fit with the experimental results obtained. As can be seen from the data in Table 4 and Figures S3 and S6, for release studies from samples based on O/W support, the dominant role plays the Korsmeyer–Peppas model, for which the fit expressed as the R^2^ value is the highest and ranges between 0.9985 and 0.9665. Moreover, *n* values of all formulations following the Korsmeyer–Peppas model are within the range of 0.6650 to 0.8879, indicating non-Fickian diffusion [62]. This semiempirical model relates exponentially the drug release to the elapsed time, and it is used to evaluate the release of the active substance when the release mechanism is not well recognized or when more than one type of release phenomenon can be involved. However, the Hixson–Crowell model also shows a high degree of fit, which is particularly evident in the release of asiaticoside. According to this model, the rate of release of active substances is limited by the dissolution rate rather than the diffusion through the matrix. Since asiaticoside has distinctly better solubility in non-polar systems, encapsulation in an O/W emulsion gives its higher concentration in the inner phase of the carrier. Thus, its release requires first entering the external phase of the emulsion, in which the solubility of asiaticoside is lower. Only then will the substance be able to be released from the topical formulation at the target site. In general, the Hixson–Crowell model applies to systems where the dissolution occurs parallel to the surface and the carrier’s dimensions diminish proportionally while keeping constant the initial geometrical form throughout all the release tests. A plot of the cubic root of the unreleased fraction of active substance vs. time will be linear if the equilibrium conditions are not reached and if the geometrical shape of the carrier form diminishes proportionally over time [63]. It can be assumed that an O/W emulsion, the outer phase of which is composed of hydrophilic substances, can partially degrade during dissolution tests. Hence the better fit of the Hixson–Crowell model in the case of asiaticoside release, especially when it was introduced into the carrier at a higher concentration (Table 4). These results are in good agreement with the report of Matos et al., where the release of active compounds from *Acanthus mollis* L. leaf extract embedded in oil-in-water topical formulation was investigated [64].

Evaluating the results of the controlled release of madecassoside and asiaticoside from W/O emulsion as support (Table 5), it can be concluded that each of the active substances follows a different kinetic model. Madecassoside release pattern gives a good fit with the Korsmeyer–Peppas model (Figure S4), especially when a higher concentration of this substance is introduced (Figure S7). Values of the *n* parameter reach 0.6024 and indicate anomalous transport of madecassoside, which is released according to the non-Fickian diffusion [62]. Moreover, due to the localization of madecassoside in the deeper parts of the carrier, the effect of the concentration of the active ingredient on the release kinetics is clearly visible. Due to its better solubility in water, madecassoside is encapsulated mostly in droplets of the dispersed phase. Therefore, its amount penetrating into the external phase of the carrier at lower concentrations makes that even for the best kinetic model (Korsmeyer–Peppas), the R^2^ correlation parameter reaches only 0.9527 and 0.9005 for tests conducted at an acceptor medium with pH = 5.8 and pH = 7.4, respectively. Nevertheless, as the loading of the carrier with the active substance increases to 5 wt.% of madecassoside, the fitting parameter R^2^ exceeds 0.9974 (Table 5). Also, values of the *n* parameter increase and may indicate the anomalous diffusion, which is controlled by more than one process. On the contrary, for asiaticoside release from W/O emulsion, a good fit with the Higuchi model can be observed, which proves that the release process is controlled by the diffusion of active substances through the carrier. Based on a pseudo-steady-state approach, a direct proportionality between the cumulative amount of the substance released and the square root of time can be plotted (Figures S3 and S6). [65]. Taking into account the good solubility of asiaticoside in the non-polar systems, the outer phase of the emulsion acts as a reservoir with prolonged release toward the acceptor fluid and reaches a pseudo-infinite dose release model. This observation is in good correlation with previously obtained results for active substances released from emulsion-based systems [59,66,67].

In the case of the gel-based carrier, kinetic parameters follow a pattern similar to that observed for samples based on the O/W emulsion. For madecassoside release studies, the Korsmeyer–Peppas model plays the dominant role, as the fit expressed as the R^2^ value is the highest and ranges between 0.9974 and 0.9823. Also, *n* values are within the range of 0.5670 to 0.7148, indicating non-Fickian diffusion (Table 6). However, the Hixson–Crowell model can also be considered, as it specifically addresses the fact that the surface of a carrier often changes with time. This can be likely observed in a hydrogel-based carrier supported by natural polymers. The use of this type of carrier in an aqueous acceptor medium that easily penetrates and erodes the polymeric matrix makes it easy to release the substance in the case of water-soluble madecassoside. Moreover, it can be assumed that the release from the carrier to the acceptor medium is fast, and the whole process is controlled mainly by diffusion through the matrix [64]. On the contrary, in the case of asiaticoside, whose solubility in aqueous media is lower, the penetration of the acceptor medium deep into the matrix and its swelling begin to play a key role, as the diffusion through the matrix becomes the main parameter controlling the release of the active substance [68]. Therefore, the Hixson–Crowell is the best model describing the release of asiaticoside from the gel-based topical formulation (Figures S5 and S8).

### 2.3. Stability of Madecassoside and Asiaticoside in W/O Emulsion, O/W Emulsion and Gel

**Figure 5:**
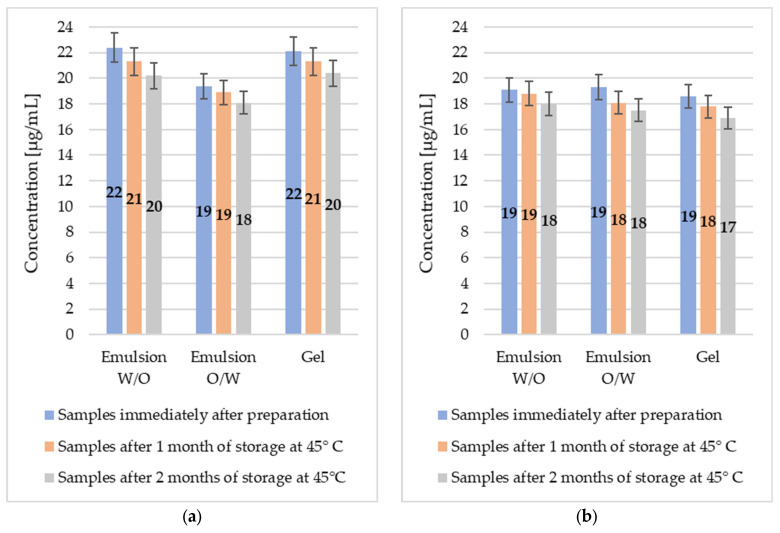
Results of quantitative determination of madecassoside (**a**) and asiaticoside (**b**) of *Centella asiatica* L. origin, introduced to the sustainable topical formulations.

Accelerated stability testing enables not only the estimation of the shelf life of the cosmetic products but also the evaluation of active substances thermal stability. Results of HPLC-based determination of madecassoside and asiaticoside content remaining in the different formulations kept at the elevated temperature are depicted in Figure 5. It shows moderate variations of madecassoside and asiaticoside amounts for the conditions studied (2 months, 45 °C). The concentrations of madecassoside and asiaticoside remained almost constant over time, proving that the carriers applied were effective in protecting the active substance.

Our findings are similar to the result of the previous work of Gomes et al., who investigated the stability of *Centella asiatica* L. extract entrapped in the polymeric colloidal nanocarriers. They found that madecassoside concentration did not change significantly during 60 days of storage in the evaluated temperature (40 °C) [69]. These results are important also in the context of studies indicating low thermal stability of the tested active substances, for which higher concentrations are obtained in the case of extractions of *Centella asiatica* L. conducted at lower temperatures [70]. On the other hand, Monton et al., who tested stability of madecassoside and asiaticoside in film-forming polymeric dispersions, observed high fluctuations of madecassoside and asiaticoside content in the formulations stored at 40 °C [71]. Similar observations were made by Puttarak et al. during stability investigations of the standardized centelloids-enriched *Centella asiatica* L. extract. They found that the amount of madecassoside and asiaticoside was decreased by approximately 20% within twelve weeks of storage under light-protected conditions. Additionally, increased storage temperature substantially accelerated the degradation process [60].

## 3. Materials and Methods

### 3.1. Chemicals

#### 3.1.1. Active Substances Present in Emulsions and Gel

A highly refined triterpenoid saponin from *Centella asiatica* L., namely madecassoside (trade name: TEGO^®^ Natural Madecassoside) and asiaticoside (trade name: Asiaticoside^®^), were obtained from Evonik Operations GmbH, Essen, Germany, and Givaudan International SA, Kempttal, Switzerland, respectively. *Centella asiatica* L. extract with a trade name CICA EX (INCI: Butylene Glycol, Aqua, *Centella asiatica* L. Extract, Madecassoside, Asiaticoside) used in the determination of madecassoside and asiaticoside stability in W/O and O/W emulsions and gel was produced by RADIANT, Chuncheon, Republic of Korea, and obtained from DKSH, Warsaw, Poland.

#### 3.1.2. Raw Materials Used to Obtain Oil-in-Water (O/W) Emulsion

The non-ionic emulsifier Galehemp OW (INCI: Polyglyceryl 4 Hemp Seedate, Caprylyl Capryl Glucoside, Aqua) was produced by Gale & Cosm S.r.l. (Bollate, Italy) and supplied by Aston Chemicals Ltd. (Aylesbury, UK). Instathix^®^ (INCI: Xanthan Gum, Sodium Stearoyl Lactylate, Tapioca Starch, Algin) was produced by Alchemy (Ascot, Berkshire, England) and supplied by IMPAG Chemicals Poland Sp. z o. o. (Warsaw, Poland). HyaCare^®^ Tremella (INCI: Tremella Fuciformis Sporocarp Extract) was produced by Evonik Operations GmbH and supplied by PPU Adara Sp. z o. o. (Warsaw, Poland). Natural origin emollients: Phytosqualan^®^ (INCI: Squalane) and Synovea^®^ EL (INCI: Ethyl Linoleate, Ethyl Palmitate, Ethyl Oleate, and Ethyl Linolenate) were produced by Biesterfeld Spezialchemie GmbH (Köln-Rodenkirchen, Germany) and supplied by Biesterfeld Chemia Specjalna Sp. z o. o. (Warsaw, Poland). LIPEX^®^ PreAct™ (INCI: Canola Oil) and Akogel™ (INCI: Hydrogenated Vegetable Oil) were supplied by Nordmann, Rassmann GmbH (Hamburg, Germany). Raspberry Necta^®^ (INCI: Rubus Idaeus Seed Oil) and CeraFluid^®^ (INCI: Triolein, Glyceryl Dioleate, Ceramide NP) were supplied by Amita Health Care, Warszawa, Poland. Citropol H (INCI: Polycitronellol) and E-Leen Green C (INCI: Pentylene Glycol, Glyceryl Caprylate/Caprate) were supplied by Safic-Alcan, Warszawa, Poland. Tocomix L70-IP Organic CO (INCI: Tocopherol, Helianthus Annuus Seed Oil) and ZEMEA^®^ Propanediol (INCI: Propanediol) were supplied by IMCD Group (Rotterdam, The Netherlands). Ceramidone^®^ (INCI: Octyldodecyl PCA) was produced by Solabia Group (Ille-de-France, France) and supplied by Aston Chemicals Ltd. All reagents were of analytical purity and were used without any pretreatment. The aqueous phase was prepared using distilled water.

#### 3.1.3. Raw Materials Used to Obtain Water-in-Oil (W/O) Emulsion

The non-ionic emulsifier, EWO Vegetable Emulsifier (INCI: Glyceryl Oleate, Polyglyceryl-3-Polyricinoleate, Olea Europea Oil Unsaponifiables), was produced by EfpBiotek (Figueira da Foz, Portugal) and supplied by IMPAG Chemicals Poland Sp. z o. o. Natural origin emollients: Softigen^®^Pura (INCI: Caprylic/Capric Triglyceride, Hydrogenated Rapeseed Oil), Phytosqualan^®^ (INCI: Squalane), and Synovea^®^ EL (INCI: Ethyl Linoleate, Ethyl Palmitate, Ethyl Oleate, and Ethyl Linolenate) were supplied by Biesterfeld Spezialchemie GmbH. Natural oils: LIPEX^®^ Omega 3/6™ (INCI: Olus Oil, Camelina Sativa Seed Oil), LIPEX^®^ PreAct™ (INCI: Canola Oil) and Akogel™ (INCI: Hydrogenated Vegetable Oil) were produced by AAK Sweden AB (Malmö, Sweden) and supplied by Nordmann, Rassmann GmbH. Natural oil Raspberry Necta^®^ (INCI: Rubus Idaeus Seed Oil), emollient Emotion^®^ Light (INCI: Tripelargonin), and a mix of ceramide and triolein—CeraFluid^®^ (INCI: Triolein, Glyceryl Dioleate, and Ceramide NP) were supplied by Amita Health Care Poland. Ceramidone^®^ (INCI: Octyldodecyl PCA) was produced by Solabia Group and supplied by Aston Chemicals Ltd. Tocomix L70-IP Organic CO (INCI: Tocopherol, Helianthus Annuus Seed Oil) and ZEMEA^®^ Propanediol (INCI: Propanediol) were supplied by IMCD Group. RonaCare^®^ Magnesium Sulphate (INCI: Magnesium Sulfate) was produced and supplied by Merck (Rahway, NJ, USA) and supplied by Barentz Sp. z o. o. (Warsaw, Poland). E-Leen Green C (INCI: Pentylene Glycol, Glyceryl Caprylate/Caprate) was supplied by Safic-Alcan Poland. All reagents were of analytical purity and were used without any pretreatment. The aqueous phase was prepared using distilled water.

#### 3.1.4. Raw Materials Used to Obtain Gel (G)

Instathix^®^ thickener (INCI: Xanthan Gum, Sodium Stearoyl Lactylate, Tapioca Starch, Algin) was produced by Alchemy and obtained from IMPAG Chemicals Poland Sp. z o. o. ZEMEA^®^ Propanediol (INCI: Propanediol) was supplied by IMCD Group. E-Leen Green C (INCI: Pentylene Glycol, Glyceryl Caprylate/Caprate) was supplied by Safic-Alcan Poland. All reagents were of analytical purity and were used without any pretreatment. The aqueous phase was prepared using distilled water.

#### 3.1.5. Chemicals Used in the Controlled Release Tests and HPLC Analysis

Ethanol 99.8% was supplied by POCh, Gliwice, Poland. Analytical standards of madecassoside (98.0%) and asiaticoside (98.0%) were supplied by Biopurify Phytochemicals Ltd., Chengdu, China. HPLC-grade trifluoroacetic acid and acetonitrile were supplied by Honeywell Chemicals (Charlotte, NC, USA) and Merck KGaA (Darmstadt, Germany), respectively. Buffer solutions with pH 5.80 and pH 7.40 were obtained from Honeywell Chemicals.

### 3.2. Preparation of Cold-Processed Emulsions and Gel

O/W emulsion was prepared at room temperature (25 °C) by addition of the oil phase containing emulsifier to the water phase containing a specified amount (2 wt.% or 5 wt.%) of madecassoside and asiaticoside. The process was performed under continuous stirring with a Hei-Torque 100 Value stirrer (Heidolph Scientific Products GmbH, Schwabach, Germany), followed by homogenization with an IKA Ultra Turrax T 18 Digital (IKA Werke GmbH & Co. KG, Staufen, Germany). The synthesis resulted in samples labeled as 2% A + M—W/O and 5% A + M—W/O, respectively.

W/O emulsion was prepared at room temperature (25 °C) by stepwise addition of the water phase containing a specified amount (2 wt.% or 5 wt.%) of madecassoside and asiaticoside to the oil phase containing the emulsifier under continuous stirring using a Hei-Torque 100 Value stirrer. The resulting mixture was homogenized using an IKA Ultra Turrax T 18 Digital homogenizer. The synthesis resulted in samples labeled as 2% A + M—O/W and 5% A + M—O/W, respectively.

Cosmetic gel was prepared at 25 °C by the addition of thickener to the water phase containing preservative and 2 wt.% or 5 wt.% of madecassoside and asiaticoside. The resulting mixture was kept under continuous stirring with a Hei-Torque 100 Value stirrer until a homogeneous gel was obtained. The synthesis resulted in samples labeled as 2% A + M—G and 5% A + M—G, respectively.

### 3.3. Physicochemical Properties and Stability Test of Cold-Processed Cosmetic Emulsions and Gel

The initial physicochemical properties, namely pH value, viscosity, and density of the emulsions and gel with 10 wt.% of *Centella asiatica* L. extract, were investigated. The pH values were assessed with a Fisherbrand™ Accumet AB 150 pH-meter (Fisher Scientific, Waltham, MA, USA). Viscosity was evaluated using a DV2T viscometer (AMETEK Brookfield, Middleboro, MA, USA) equipped with a small sample adapter. The spindle no. SC4-29 (for W/O emulsion) and the spindle no. SC4-21 (for O/W emulsion and gel) were used. Density was measured using a P-1 metal pycnometer (Pol-Zaf s.c., Wrocław, Poland). All the measurements were done in triplicate, and the averaged values with SD (*n* = 3) were reported. Additionally, parameters such as color, smell, and appearance of the cosmetic formulations were also evaluated organoleptically.

Stability of the cosmetic formulations was investigated by a variety of techniques, such as laser diffraction (LD), electrophoretic light scattering (ELS), and static multiple light scattering (SMLS). All of the tests were conducted directly after the preparation of the samples as well as after their incubation for 2–3 months at 45 °C, regarding both pure cosmetic carriers and their counterparts containing active substances, namely madecassoside and asiaticoside.

The particle size distributions of the emulsions were analyzed by means of LD technique using Mastersizer 2000 apparatus (Malvern Panalytical Ltd., Malvern, UK) with a wet dispersion unit. For each sample, the measurements were repeated five times. Before the analysis, the refractive index of each sample was determined by using the Refracto™ 30GS portable refractometer (Mettler Toledo, Greifensee, Switzerland).

Zeta potential was quantified using a Zetasizer Nano-ZS analyzer (Malvern Panalytical Ltd., UK), which operates under the ELS technique. Prior to the measurement, 0.05 g of emulsion or gel was suspended in 5 g of distilled water by using an ultrasonic bath, and the resulting mixture was further diluted (60 µL of emulsions or 120 µL of gel in 15 mL of H_2_O) to avoid multiple scattering effects. The samples were then placed in the suitable PS cuvettes and subjected to ELS measurements at 25 °C using the Smoluchowski model. The resulting values (*n* = 10) were then averaged, and the standard deviation was calculated.

The Turbiscan^LAB^ Expert device (Formulaction, Toulouse, France) was used to evaluate the backscattering (ΔBS) and transmission (ΔT) profiles of emulsions and gel-based formulations as a function of incubation time at elevated temperature (45 °C). The samples tested were placed directly into calibrated borosilicated cylindrical glass vials with stoppers and scanned from bottom to top for a total of 5 min, with one scan at every 30 s (*n* = 10). The obtained data were processed by a Turby Soft 2.0 software and reported as Turbiscan Stability Index (TSI) vs. time. TSI is a statistical parameter that can be used to estimate the stability of samples. It is expressed by the following Equation (1):

(1) $$TSI\left(t\right)=\frac{1}{N_{h}}\sum_{t_{1}=1}^{t_{max}}\sum_{z_{i}=z_{min}}^{z_{max}}\left|BST\left(t_{i},z_{i}\right)-BST(t_{i-1},z_{i})\right|$$

where: *N_h_* = [(*z_max_* − *z_min_*)/Δ*h*]—the number of height positions in the selected zone of the scan; *t_max_*—the measurement point corresponding to the time t at which the *TSI* is calculated; *z_min_* and *z_max_*—the lower and upper selected height limits, respectively; *BST*—the considered signal (BS if T < 0.2%, T otherwise) [72].

### 3.4. Stability Test of Madecassoside and Asiaticoside in O/W and W/O Emulsion and Gel

The stability test of madecassoside and asiaticoside in W/O and O/W emulsions and gel involved the incorporation of *Centella asiatica* L. extract containing the active substances into the samples of both types of emulsions and gel. 10 wt.% of *Centella asiatica* L. extract has been added to the water phase of O/W and W/O emulsions and gel during their preparation, as it has been described in Section 2.2. The formulations obtained were used for the determination of madecassoside and asiaticoside content by HPLC method (see Section 3.6) immediately after preparing the carriers and after 1 and 2 months of storing the samples in the elevated temperature (45 °C). Samples for the stability test of madecassoside and asiaticoside in the cosmetic supports were prepared by the extraction method using 1.0 g of O/W, W/O emulsions, or gel containing *Centella asiatica* L. extract and 10 mL of ethanol. Each sample was shaken manually for 10 min and then treated in the ultrasonic bath for 15 min. The resulting solution was filtered several times through a PTFE syringe filter with a pore size of 0.22 μm. Then 1.5 mL of clear solution was transferred to a glass vial for HPLC analysis.

### 3.5. Controlled Release of Madecassoside and Asiaticoside

The in vitro release study of madecassoside and asiaticoside from the W/O and O/W emulsion and gel as carriers was conducted with a Varian Vankel 7010 apparatus (Agilent Technologies, Santa Clara, CA, USA). The formulations, prepared as described in Section 2.2, contained 2 wt.% of madecassoside and 2 wt.% of asiaticoside, or 5 wt.% of madecassoside and 5 wt.% of asiaticoside. The experiments were performed using flow-through cells and a membrane made of regenerated cellulose (Cuprophan^®^, Medicell International Ltd., London, UK). The membrane was soaked for 10 min in a respective buffer solution (pH = 5.8 or pH = 7.4) before use. Approximately 1 g of the tested formulation was placed in the diffusion cell and fitted with a cellulose membrane, followed by placing the diffusion cell in the acceptor chambers filled with 100 mL of the acceptor medium. Phosphate and borate buffers with pH values of 5.8 and 7.4, respectively, stirred at 100 rpm, were used as acceptor fluids with temperature maintained at 37 °C ± 0.5 °C. The release tests were carried out for 24 h with 1 mL sample of acceptor medium taken every 15 min (during the first 2 h) and every 60 min (during the next 6 h). The last sample was taken after 24 h. After each sampling, the acceptor medium was immediately supplemented with 1 mL of the fresh buffer solution. The sampled acceptor medium was filtered through a syringe PTFE filter with a pore diameter of 0.22 μm and transferred to a glass vial for HPLC analysis. The concentrations of madecassoside and asiaticoside were determined according to the procedure described in Section 3.6, and the release profiles were constructed. Each formulation was duplicated in order to check the reproducibility of the release test, and the obtained results were averaged.

To determine the release mechanism of madecassoside and asiaticoside from the synthesized topical formulations, selected kinetic models were investigated by applying Equations (2)–(5):

(2) $$First\;order\;kinetics\;log\frac{Q_{t}}{Q_{0}}=\frac{K_{1}}{2.303}\cdot t$$

(3) $$\mathrm{Higuchi}\;\mathrm{model}\;\frac{Q_{t}}{Q_{0}}=K_{H}\sqrt{t}$$

(4) $$Korsmeyer–Peppasmodel\;log\frac{Q_{t}}{Q_{0}}=n\cdot logt+logK_{K}$$

(5) $$\mathrm{Hixson}\text{–}\mathrm{Crowell}\;\mathrm{model}\;{\left(1-\frac{Q_{t}}{Q_{0}}\right)}^{\frac{1}{3}}={1-K}_{\beta }\cdot t$$

*Q_t_*—amount of the active substance released from the carrier, mg/mL

*Q*_0_—initial amount of the active substance on the carrier, mg/mL

*t*—duration of the release process, min

*K*_1_, *K_H_*, *K_K_*, *K_β_*—coefficients specific to the particular kinetic model.

### 3.6. Determination of Madecassoside and Asiaticoside by HPLC Analysis

#### 3.6.1. Chromatographic Conditions

Qualitative and quantitative determination of the madecassoside and asiaticoside was performed using a Varian 920-LC high-performance liquid chromatograph (Agilent Technologies, USA) equipped with an automatic sample changer and UV-VIS detector working at the wavelength of 205 nm. A 0.1 wt.% solution of trifluoroacetic acid (TFA) in water and acetonitrile in the volumetric ratio of 72:28 was used as a mobile phase, with an isocratic flow of 1.0 mL/min. The amount of 20 μL of each sample was injected into the TSK gel C18 column (250 × 4.6 mm, with 5 µm width of the film). Each of the samples tested was subjected to three injections, and the arithmetic mean was calculated from the results obtained. The concentrations of madecassoside and asiaticoside in the samples were estimated from the calibration standard curve.

#### 3.6.2. Preparation of Standard Solutions

A stock solution of madecassoside and asiaticoside at a concentration of 0.1 mg/mL was prepared in a 50-mL volumetric flask by using ethanol as a solvent. Madecassoside and asiaticoside standard solutions of 0.08, 0.07, 0.06, 0.05, 0.04, and 0.03 mg/mL were then prepared from the stock solution by successive dilutions in the 10 mL volumetric flask. The standard solutions were filtered through a 0.45 µm pore-size PTFE syringe filter and analyzed by the HPLC instrument in triplicate for each injection (*n* = 3). The calibration curves of madecassoside and asiaticoside with R^2^ coefficients equal to 0.999 were prepared by plotting the peak area against the concentration of madecassoside and asiaticoside standards.

#### 3.6.3. Method Validation

The blank sample was analyzed by an HPLC instrument (*n* = 6). LOD and LOQ were calculated according to Equations (6) and (7), using a standard deviation of the peak area of the blank sample (B_STD_) and the slope of the calibration curve (S).

LOD = (3.3 × B_STD_)/S (6)

LOQ = (10 × B_STD_)/S (7)

Precision was calculated using the relative standard deviation from HPLC analysis (*n* = 3) of standard solutions with concentrations of 0.050, 0.070, and 0.090 mg/mL. It was reported as the percent relative standard deviation (% RSD) of the analysis. The recovery method was used to calculate accuracy by spiking *Centella asiatica* L. extract with madecassoside and asiaticoside standards at concentrations of 0.050, 0.070, and 0.090 mg/mL. The samples obtained were then analyzed (*n* = 3) by the HPLC instrument, and the percent of recovery was reported.

## 4. Summary

During our study, sustainable cold-processed topical formulations supporting the treatment of atopic dermatitis were obtained. Different types of carriers were designed, taking into account the needs of atopic skin. The W/O emulsion contained the largest amount of emollients (31%); the O/W emulsion contained 21% of emollients, whereas the gel did not contain emollients. All carriers were obtained with carefully selected sustainable raw materials and by using low-energy processes that eliminate heating of the components. Stability tests showed that the formulations obtained had good stability, especially the O/W system, which showed no major changes in the particle size of the dispersed phase even after 84 days of storage at elevated temperatures. For emulsions obtained by the cold process, obtaining good stability is difficult due to the limited number of emulsifiers operating at ambient temperatures. Nevertheless, low temperature supports the stability of the active substances introduced, as our accelerated stability tests showed that madecassoside and asiaticoside introduced into cold-processed formulations were characterized by good stability even during storage at elevated temperatures. In addition, factors influencing release profiles from different carriers, such as active substance concentration and pH of the acceptor medium, have been tested, considering the physiology of atopic skin and its needs. The pH values of acceptor media were selected in relation to the pH values observed in the healthy skin (pH = 5.8) as well as the skin with atopic dermatitis (pH = 7.4). The results showed that the pH value of the acceptor medium did not play a significant role during the release study of madecassoside. In contrast, it becomes apparent in the case of asiaticoside release. However, such complementarity can be a major advantage for products designed for atopic skin, in which pH is disturbed. Moreover, regardless of the type of carrier, increasing the concentration of active ingredients from 2 wt.% to 5 wt.% did not affect the cumulative release of madecassoside and asiaticoside. The largest amounts of madecassoside were released from carriers with a large amount of hydrophilic phase, as the amount of lipid-based emollients present in the formulation regulates the viscosity of the carrier and consequently influences the release profiles. The varied physicochemical properties of the carriers and the active substances caused that the release profiles followed different mechanisms, indicating that more than one type of release phenomenon can be involved. Our study shows that the kinetics and release profiles of triterpenoids from *Centella asiatica* L. may provide important information on the most optimal type of carrier, the pH of the medium, and the concentration of the active substance. Furthermore, they indicate that the synthesized formulations can be successfully used as carriers of triterpenoids present in *Centella asiatica* L. extract. Furthermore, it is important to point out that obtaining madecassoside and asiaticoside carriers in a low-temperature process undoubtedly promotes the durability of both the active substances and the lipid-based substances (emollients) used, of which a significant amount is advisable for products intended for atopic skin.

## Figures and Tables

**Table 1:** Sustainability parameters of raw materials used in formulations (based on Material Safety Data Sheets and documentation shared by producers of raw materials).

Trade Name of Raw Material	Origin of Ingredients	Green Technology	Food Waste Origin	Bio-Degradability	Toxicity to Aquatic Organisms
TEGO^®^ Natural Madecassoside	Vegetable: *Centella asiatica* L. leaves	Extraction and purification of *Centella asiatica* L.	---	No data available	No data available
Asiaticoside^®^	*Centella asiatica* L. leaves	Extraction, drying	---	No data available	No data available
EWO—Vegetable Emulsifier	Olive oil fatty acids	Olive oil fatty acids collected from olive oil refineries and purified into olive oil unsaponificable	Olives from industry by-products	Readily biodegradable: 69–95% in 28 days (OECD 301B).	No data available
Softigen^®^Pura	Vegetable: seeds from rapeseed, palm kernel oil or seed coconut oil	Refined, blanched, deodorized	---	No data available	No data available
Phytosqualan^®^	Vegetable: olives	Esterification, distillation, saponification, hydrogenation, winterization	Olives from food waste	Readily biodegradable: 67% in 28 days (OECD 301B)	No data available
Synovea^®^ EL	Vegetable: safflower seed oil	Green esterification	---	Readily biodegradable: 100% in 28 days (OECD 301B)	No data available
LIPEX^®^ Omega 3/6™	Vegetable oils	Mechanical extraction, refining, deodorizing	---	Readily biodegradable (OECD 301B)	Non-toxic to freshwater algae and low acute aquatic toxicity
LIPEX^®^ PreAct™	Vegetable: seeds from rapeseed	Mechanical pressing, deodorizing, hydrogenation, filtration, solvent extraction	---	Readily biodegradable (OECD 301B)	Non-toxic to freshwater algae and low acute aquatic toxicity
Akogel™	Vegetable: fruit from coconut nucifera, seeds of *Glycine max*, *Brassica napus*, and *Arecaceae*.	Mechanical extraction, solvent extraction, refining, deodorizing, hydrogenation	---	Readily biodegradable (OECD 301F)	Non-toxic to freshwater algae and low acute aquatic toxicity
Raspberry Necta^®^	Vegetable: raspberries seeds	Cold-processed extraction	Raspberries pulp as a waste product of the juice production industry.	Readily biodegradable (OECD 301F)	Not determined
Emotion^®^ Light	Vegetable: *Silybum marianum* seed oil	Pure pelargonic acid: distillation and hydrolysis of *Silybum marianum* seed oil; sustainable esters: high-temperature esterification	---	Readily biodegradable: 81.23% in 28 days (OECD 301B)	Not classified for environmental hazards. Aquatic acute toxicity: EC50 (10 days) *Desmodesmus subspicatus* > 4.4 mg/L
CeraFluid^®^	Vegetable: triolein and glyceryl dioleate from *Olea europaea*; Biotechnology: Ceramide NP	Triolein and glyceryl dioleate: distillation of olive fruit oil for pure fatty acids and their esterification. Ceramide NP: amidation reaction of vegetal stearic acid on phytosphingosine, obtained through bio-fermentation process using yeasts on vegetable substrate.	Olive fruit from food waste.	Readily biodegradable (OECD 301B)	Short term toxicity to fish: LC50 (48 h) 1–10 g/L Short term toxicity to aquatic invertebrates: EL50 (48 h) 100 mg/L Long term toxicity to aquatic invertebrates: NOELR (21 days) 10 mg/L, LOELR (21 days) 10 mg/L Toxicity to aquatic algae and cyanobacteria: EL50 (72 h) 296–320 mg/L
Ceramidone^®^	Vegetable: L-glutamic acid	Cyclization of L-glutamic acid to L-pyrrolidone carboxylic acid (L-PCA); esterification of L-PCA to octyldodecyl ester.	---	Readily biodegradable (OECD 301D)	Not considered harmful to aquatic organisms; not causing long-term adverse effects on the environment: EC50 *Daphnia magna*: 2.6 mg/L; EC50 (72 h) algae: 12 mg/L.
Tocomix L70-IP Organic CO	Tocopherol: soy beans; *Helianthus annuus* seed oil: sunflower seeds	Tocopherol: separation in refining stream, purification; *Helianthus annuus* seed oil: pressing of seeds, refining of crude oil.	From a stream by-product in the refinery process of soybean oil.	Inherently biodegradable (OECD 301F)	Not considered harmful to aquatic organisms: LC50 (96 h) fish: >10 mg/L; EC50 (72 h) algae: >25.8 mg/L
ZEMEA^®^	Vegetable: industrial corn converted to technical starch and next converted to glucose	Fermentation process	---	Readily biodegradable: 70.5% in 28 days (OECD 301B)	Not considered harmful to aquatic organisms: LC 50 (96 h) fish: >9.72 mg/L
RonaCare^®^ Magnesium Sulphate	Mineral	Naturally occurring	---	Biodegradability assessment methods do not apply to inorganic substances.	LC50 (48 h) *Daphnia magna*: 720 mg/L; EC50 (18 days) *Chlorella vulgaris*: 2.7 mg/L
E-Leen Green C	Vegetal: Pentylene Glycol: sugar cane bagasse; Glyceryl Caprylate/Caprate: palm kernel oil (seeds of the fruit of the oil palm), coconut oil (fruits of the coconut palm), rapeseed oil (*Brassica napus*), sunflower oil (*Helianthus annuus*).	Pentylene glycol: steam distillation of sugarcane bagasse (*Saccharum officinarum*), catalytic hydrogenation, purification; Glyceryl Caprylate/Caprate from vegetable origin melted at 40–50 °C, added to Pentylene Glycol and stirred at ambient temperature.	---	Readily biodegradable: Pentylene Glycol: 73% within 28 days (OECD 301E); Glyceryl Caprylate/Caprate: 82.6% within 28 days (OECD 301B)	Pentylene glycol: OECD 203—LC50 (96 h) zebra fish: >1096 mg/L; EU Directive 79/831/EWG Appendix V—EC50 (48 h) *Daphnia magna*: >500 mg/L; Glyceryl Caprylate/Caprate: short-term toxicity to fish and aquatic invertebrates: no toxicity up to the limit of water solubility; toxicity to aquatic algae and cyanobacteria: ErL50 (72 h) nominal, loading rate = 49 mg/L; NOErLR (72 h), nominal, loading rate = 20.7 mg/L; NOEC (72 h), measured concentration = 1.19 mg/L
Galehemp OW	Polyglycerine-4—mixture of refined polyglycerols of vegetable origin; hemp seed oil—product extracted by mechanical pressing from the fruits of *Cannabis sativa* L.; Caprylyl/Capryl Glucoside—mild, non-ionic surfactant obtained from renewable raw materials.	Transesterification of Polyglycerine-4 and *Cannabis sativa* seed oil resulting in Cannabis sativa seed oil polyglyceryl-4 esters; mixing with caprylyl/capryl glucoside and water resulting in trans-esters between tetraglycerol and hemp seed oil.	---	Readily biodegradable (OECD 301B)	No data available
Citropol H	Raw material terpenes from Forest Stewardship Council (FSC) certified pine.	Intensified continuous etherification	Side-stream turpentine from the paper making process	Inherently biodegradable (OECD 301B)	No data available
Instathix^®^	Xanthan Gum: biotechnological process; Tapioca Starch: naturally derived from tapioca; Algin: naturally derived from brown seaweed; Sodium Stearoyl Lactylate: biotechnological processes.	Xanthan Gum: fermentation of glucose by *Xanthomonas campestris*; Tapioca Starch: physical processing of tapioca; Algin: physical extraction of brown seaweed; Sodium Stearoyl Lactylate: esterification of palm oil (RSPO Mass Balance) with biotechnologically obtained lactic acid.	---	Readily biodegradable (OECD 301B)	Low toxicity to invertebrates, fish and algae.
HyaCare^®^ Tremella	Vegetable	Extraction followed by purification of *Tremella fuciformis* sporocarp	---	No data available	No data available

**Table 2:** Physicochemical parameters of topical formulations containing 10 wt.% of *Centella asiatica* L. extract.

Color, Odor	pH	Viscosity, cP *	Density, g/mL
white to cream, homogeneous bulk, with odor characteristic to use raw materials	Emulsion O/W		
	5.45 ± 0.01	605.40 ± 4.84	0.999 ± 0.001
	Emulsion O/W + 10 wt.% of *Centella asiatica* L. extract		
	5.30 ± 0.01	394.20 ± 5.45	0.998 ± 0.001
white to cream, homogeneous bulk, with odor characteristic to use raw materials	Emulsion W/O		
	5.32 ± 0.01	6548.20 ± 13.32	1.000 ± 0.002
	Emulsion W/O + 10 wt.% of *Centella asiatica* L. extract		
	5.10 ± 0.01	4993.60 ± 15.32	1.000 ± 0.001
semi-transparent, opalescence, and homogeneous gel, without specific odor	Gel		
	4.93 ± 0.01	1230.13 ± 7.80	0.999 ± 0.001
	Gel + 10 wt.% of *Centella asiatica* L. extract		
	4.80 ± 0.02	1050.03 ± 8.60	0.999 ± 0.001

* SC4-29 with 100 rpm for W/O emulsion and SC4-21 with 100 rpm for O/W emulsion and gel.

**Table 3:** Linearity parameters, the limit of detection (LOD), the limit of quantitation (LOQ), precision, and accuracy of madecassoside and asiaticoside analysis.

	Madecassoside	Asiaticoside
Linear equation	y = 54.6823 · x − 0.0694	y = 54.0976 · x − 0.0640
R^2^ coefficient	0.9987	0.9985
Test range, mg/mL	0.03–0.10	0.03–0.10
LOD, mg/mL	0.0045	0.0053
LOQ, mg/mL	0.0136	0.0159
Precision, % RSD	0.87	1.29
Accuracy/recovery, %	104.71 ± 1.35	111.95 ± 0.98

**Table 4:** Release parameters from O/W support according to different kinetic models.

Active Substance	pH Value of the Release Medium	t_20%_, min	X_480min_, %	Kinetic Model	R^2^	Kinetic Parameters	
						K	*n*
2% M	5.8	150	48.3	First-order kinetics	0.6222	K_1_ = 0.0018	---
				Korsmeyer-Peppas	0.9957	K_K_ = 0.5780	0.7081
				Higuchi	0.9823	K_H_ = 0.0248	---
				Hixson-Crowell	0.9985	K_β_ = 0.0004	---
	7.4	200	40.0	First-order kinetics	0.6777	K_1_ = 0.0018	---
				Korsmeyer-Peppas	0.9930	K_K_ = 0.6361	0.6650
				Higuchi	0.9740	K_H_ = 0.0220	---
				Hixson-Crowell	0.9978	K_β_ = 0.0003	---
5% M	5.8	210	37.6	First-order kinetics	0.5833	K_1_ = 0.0021	---
				Korsmeyer-Peppas	0.9883	K_K_ = 0.6461	0.8047
				Higuchi	0.9790	K_H_ = 0.0512	---
				Hixson-Crowell	0.9929	K_β_ = 0.0013	---
	7.4	270	33.2	First-order kinetics	0.6221	K_1_ = 0.0021	---
				Korsmeyer-Peppas	0.9963	K_K_ = 0.5448	0.8135
				Higuchi	0.9647	K_H_ = 0.0491	---
				Hixson-Crowell	0.9766	K_β_ = 0.0013	---
2% A	5.8	750	15.2	First-order kinetics	0.5538	K_1_ = 0.0023	---
				Korsmeyer-Peppas	0.9756	K_K_ = 0.0682	0.8879
				Higuchi	0.9695	K_H_ = 0.0092	---
				Hixson-Crowell	0.9726	K_β_ = 0.0001	---
	7.4	500	19.0	First-order kinetics	0.6317	K_1_ = 0.0021	---
				Korsmeyer-Peppas	0.9853	K_K_ = 0.1462	0.7872
				Higuchi	0.9556	K_H_ = 0.0114	---
				Hixson-Crowell	0.9894	K_β_ = 0.0001	---
5% A	5.8	1440	8.8	First-order kinetics	0.5218	K_1_ = 0.0023	---
				Korsmeyer-Peppas	0.9665	K_K_ = 0.0629	0.9647
				Higuchi	0.9672	K_H_ = 0.0134	---
				Hixson-Crowell	0.9722	K_β_ = 0.0001	---
	7.4	835	12.6	First-order kinetics	0.5637	K_1_ = 0.0028	---
				Korsmeyer-Peppas	0.9709	K_K_ = 0.0474	1.0622
				Higuchi	0.9185	K_H_ = 0.0220	---
				Hixson-Crowell	0.9888	K_β_ = 0.0003	---

**Table 5:** Release parameters from W/O support according to different kinetic models.

Active Substance	pH Value of the Release Medium	t_20%_, min	X_480min_, %	Kinetic Model	R^2^	Kinetic Parameters	
						K	*n*
2% M	5.8	75	36.4	First-order kinetics	0.5762	K_1_ = 0.0009	---
				Korsmeyer-Peppas	0.9527	K_K_ = 4.4402	0.3405
				Higuchi	0.9271	K_H_ = 0.0125	---
				Hixson-Crowell	0.7724	K_β_ = 0.0001	---
	7.4	60	34.7	First-order kinetics	0.4365	K_1_ = 0.0009	---
				Korsmeyer-Peppas	0.9005	K_K_ = 3.9857	0.3656
				Higuchi	0.8756	K_H_ = 0.0121	---
				Hixson-Crowell	0.6835	K_β_ = 0.0001	---
5% M	5.8	300	27.0	First-order kinetics	0.6459	K_1_ = 0.0016	---
				Korsmeyer-Peppas	0.9985	K_K_ = 1.6466	0.6024
				Higuchi	0.9922	K_H_ = 0.0344	---
				Hixson-Crowell	0.9548	K_β_ = 0.0011	---
	7.4	350	25.3	First-order kinetics	0.6510	K_1_ = 0.0016	---
				Korsmeyer-Peppas	0.9968	K_K_ = 1.8450	0.5686
				Higuchi	0.9953	K_H_ = 0.0303	---
				Hixson-Crowell	0.9559	K_β_ = 0.0010	---
2% A	5.8	1125	14.4	First-order kinetics	0.5206	K_1_ = 0.0012	---
				Korsmeyer-Peppas	0.9484	K_K_ = 0.7303	0.4966
				Higuchi	0.9661	K_H_ = 0.0062	---
				Hixson-Crowell	0.8017	K_β_ = 0.0001	---
	7.4	480	20.0	First-order kinetics	0.4084	K_1_ = 0.0018	---
				Korsmeyer-Peppas	0.9068	K_K_ = 0.1323	0.8296
				Higuchi	0.9656	K_H_ = 0.0093	---
				Hixson-Crowell	0.8206	K_β_ = 0.0001	---
5% A	5.8	>1440	6.2	First-order kinetics	0.5044	K_1_ = 0.0023	---
				Korsmeyer-Peppas	0.9316	K_K_ = 0.0646	0.9002
				Higuchi	0.9629	K_H_ = 0.0090	---
				Hixson-Crowell	0.9762	K_β_ = 0.0001	---
	7.4	>1440	10.0	First-order kinetics	0.4741	K_1_ = 0.0021	---
				Korsmeyer-Peppas	0.9315	K_K_ = 0.1050	0.8820
				Higuchi	0.9709	K_H_ = 0.0120	---
				Hixson-Crowell	0.9168	K_β_ = 0.0001	---

**Table 6:** Release parameters from gel (G) support according to different kinetic models.

Active Substance	pH Value of the Release Medium	t_20%_, min	X_480min_, %	Kinetic Model	R^2^	Kinetic Parameters	
						K	*n*
2% M	5.8	150	44.3	First-order kinetics	0.6284	K_1_ = 0.0018	---
				Korsmeyer-Peppas	0.9924	K_K_ = 0.6789	0.6681
				Higuchi	0.9880	K_H_ = 0.0221	---
				Hixson-Crowell	0.9836	K_β_ = 0.0003	---
	7.4	200	45.0	First-order kinetics	0.6889	K_1_ = 0.0016	---
				Korsmeyer-Peppas	0.9823	K_K_ = 1.2428	0.5670
				Higuchi	0.9907	K_H_ = 0.0214	---
				Hixson-Crowell	0.9848	K_β_ = 0.0003	---
5% M	5.8	210	38.6	First-order kinetics	0.6172	K_1_ = 0.0018	---
				Korsmeyer-Peppas	0.9974	K_K_ = 1.1280	0.7148
				Higuchi	0.9867	K_H_ = 0.0500	---
				Hixson-Crowell	0.9950	K_β_ = 0.0013	---
	7.4	270	36.4	First-order kinetics	0.6368	K_1_ = 0.0018	---
				Korsmeyer-Peppas	0.9932	K_K_ = 1.2025	0.6900
				Higuchi	0.9831	K_H_ = 0.0465	---
				Hixson-Crowell	0.9873	K_β_ = 0.0013	---
2% A	5.8	750	16.5	First-order kinetics	0.5861	K_1_ = 0.0023	---
				Korsmeyer-Peppas	0.9804	K_K_ = 0.0512	0.9291
				Higuchi	0.9424	K_H_ = 0.0099	---
				Hixson-Crowell	0.9919	K_β_ = 0.0004	---
	7.4	500	20.5	First-order kinetics	0.5873	K_1_ = 0.0020	---
				Korsmeyer-Peppas	0.9817	K_K_ = 0.1107	0.8410
				Higuchi	0.9569	K_H_ = 0.0116	---
				Hixson-Crowell	0.9857	K_β_ = 0.0001	---
5% A	5.8	1440	7.7	First-order kinetics	0.4737	K_1_ = 0.0028	---
				Korsmeyer-Peppas	0.9165	K_K_ = 0.0182	1.1399
				Higuchi	0.9690	K_H_ = 0.0114	---
				Hixson-Crowell	0.9548	K_β_ = 0.0001	---
	7.4	835	12.6	First-order kinetics	0.5165	K_1_ = 0.0023	---
				Korsmeyer-Peppas	0.9406	K_K_ = 0.0896	0.9483
				Higuchi	0.9637	K_H_ = 0.0176	---
				Hixson-Crowell	0.9924	K_β_ = 0.0002	---

## Data Availability

Dataset available on request from the authors.

## Supplementary Materials

The following supporting information can be downloaded at: https://www.mdpi.com/article/10.3390/molecules29235583/s1, Figure S1: Photographs of the prepared topical formulations containing 10 wt.% of *Centella asiatica* L. extract, Figure S2: Calibration curves for HPLC analysis of madecassoside (A) and asiaticoside (B), Figure S3: Graphic representation of kinetic models applied to the release profiles from 2% M + A—O/W sample, Figure S4: Graphic representation of kinetic models applied to the release profiles from 2% M + A—W/O sample, Figure S5: Graphic representation of kinetic models applied to the release profiles from 2% M + A—G sample, Figure S6: Graphic representation of kinetic models applied to the release profiles from 5% M + A—O/W sample, Figure S7: Graphic representation of kinetic models applied to the release profiles from 5% M + A—W/O sample, Figure S8: Graphic representation of kinetic models applied to the release profiles from 5% M + A—G sample.
