# Supplementary material for: Controlled Release of Madecassoside and Asiaticoside of Centella asiatica L. Origin from Sustainable Cold-Processed Topical Formulations
Source: Molecules. 2024 Nov 26;29(23):5583. doi: 10.3390/molecules29235583 (PMC11643272; doi:10.3390/molecules29235583)
Supplement: Supplementary file 1 [file molecules-29-05583-s001.zip › molecules-3297982-supplementary.pdf]

Controlled Release of Madecassoside and Asiaticoside of *Centella asiatica* L. Origin from Sustainable Cold-Processed Topical Formulations

Monika Krzyżostan [1,2], Agata Wawrzyńczak [1,*] and Izabela Nowak [1,*]

[1] Faculty of Chemistry, Adam Mickiewicz University in Poznań, Uniwersytetu Poznańskiego 8, 61-614 Poznań, Poland; monika.krzyzostan@amu.edu.pl
[2] Dr Koziej Instytut Badań Kosmetyków, Czerniakowska 58, 00-717 Warsaw, Poland
* Correspondence: agata.wawrzynczak@amu.edu.pl (A.W.), nowakiza@amu.edu.pl (I.N.)

Supporting Information

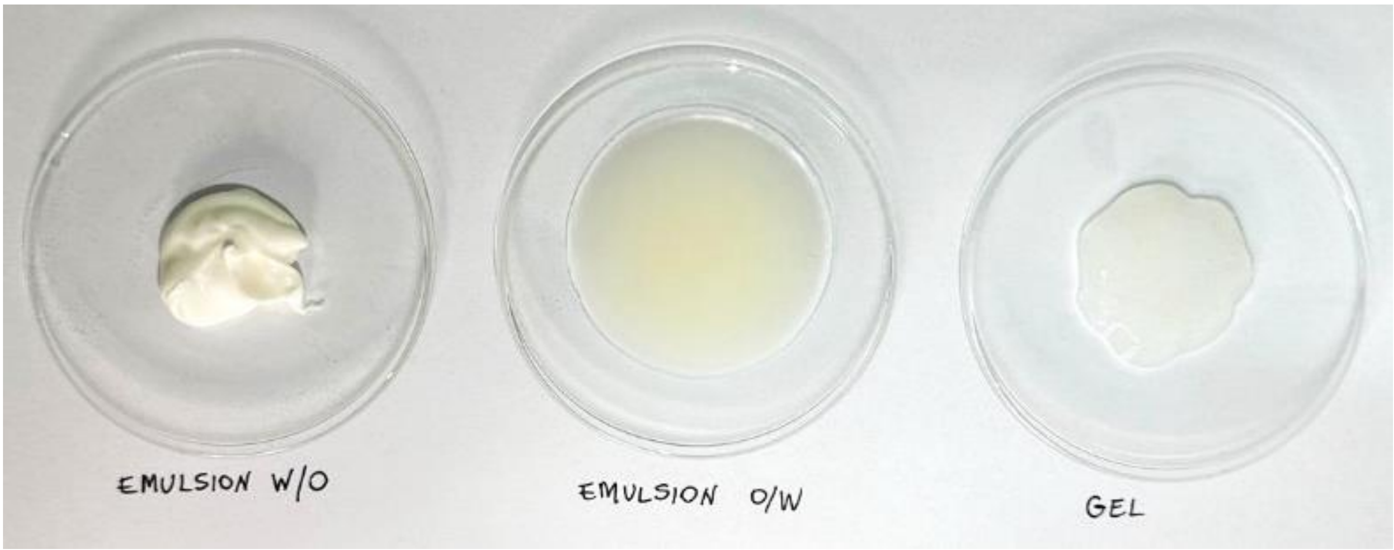

Figure S1. Photographs of the prepared topical formulations containing 10 wt.% of Centella asiatica L. extract.

A

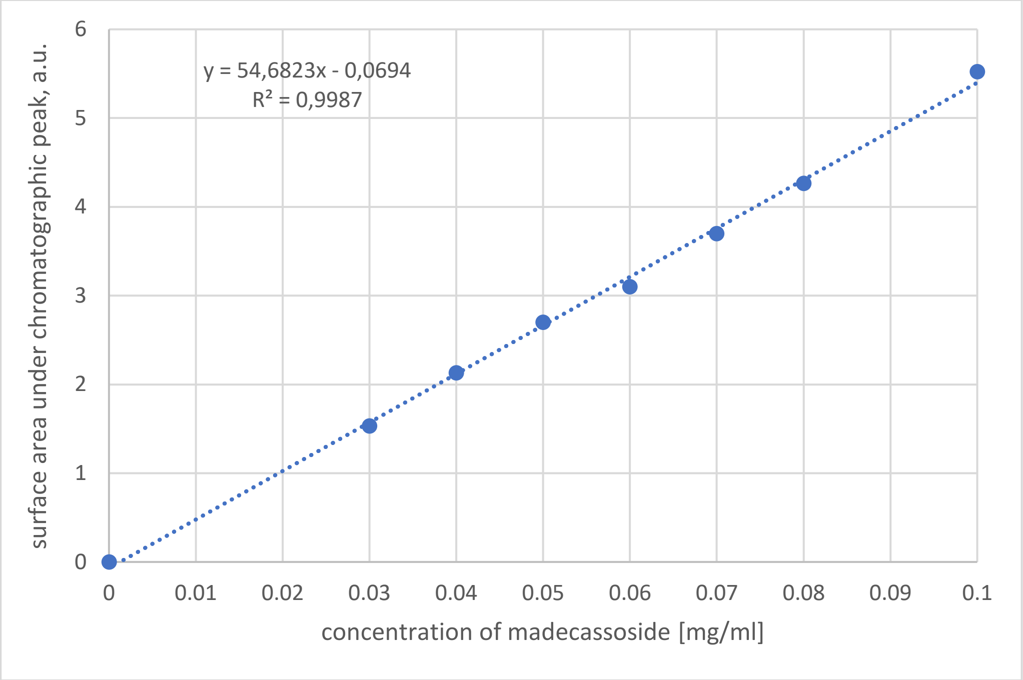

B

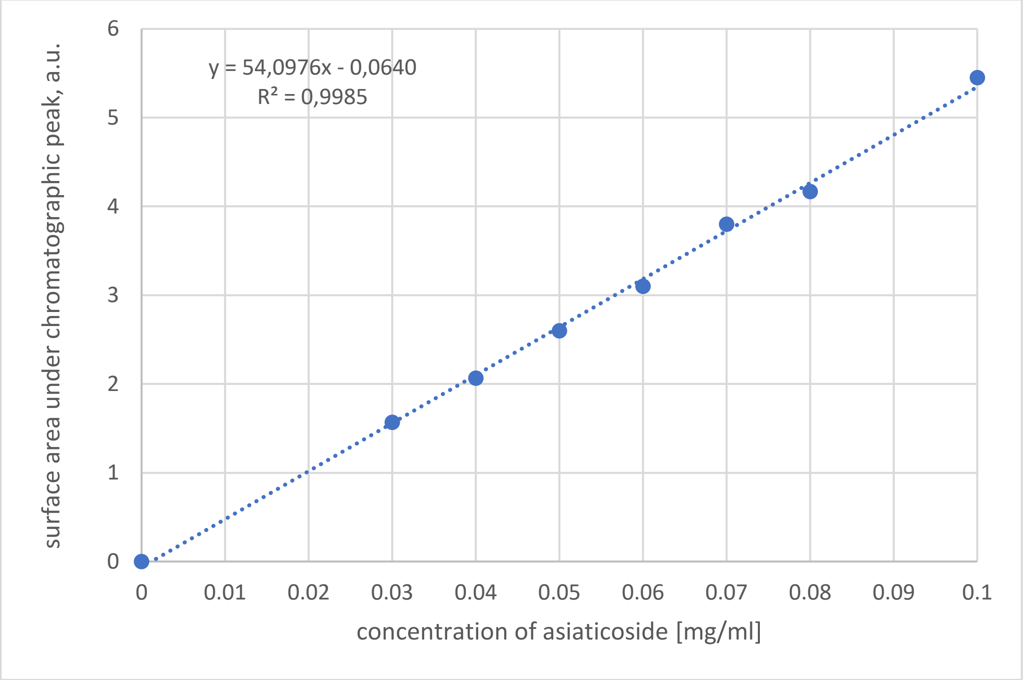

Figure S2. Calibration curves for HPLC analysis of madecassoside (A) and asiaticoside (B).

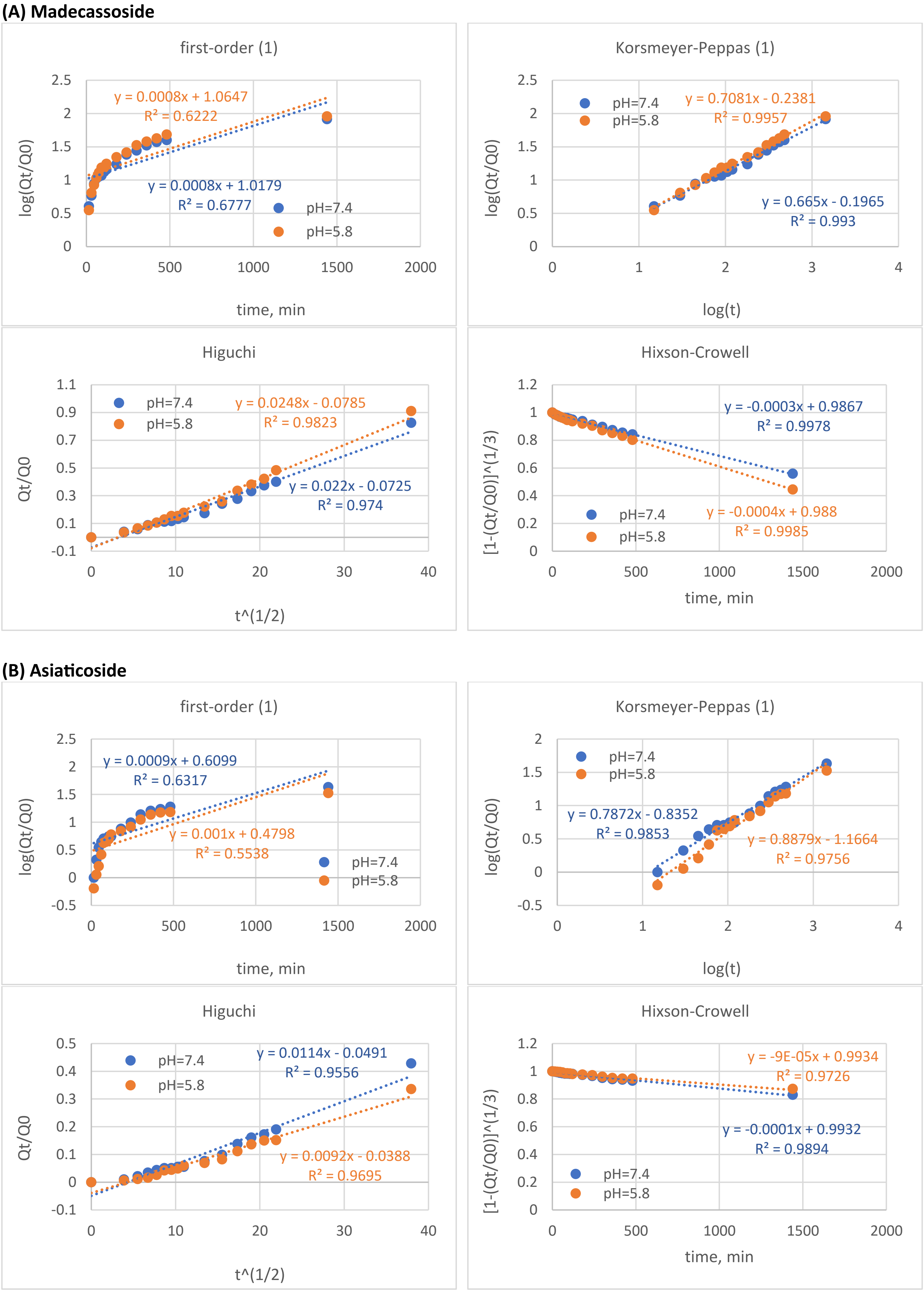

Figure S3. Graphic representation of kinetic models applied to the release profiles from 2 % M+A – O/W sample.

(A) Madecassoside

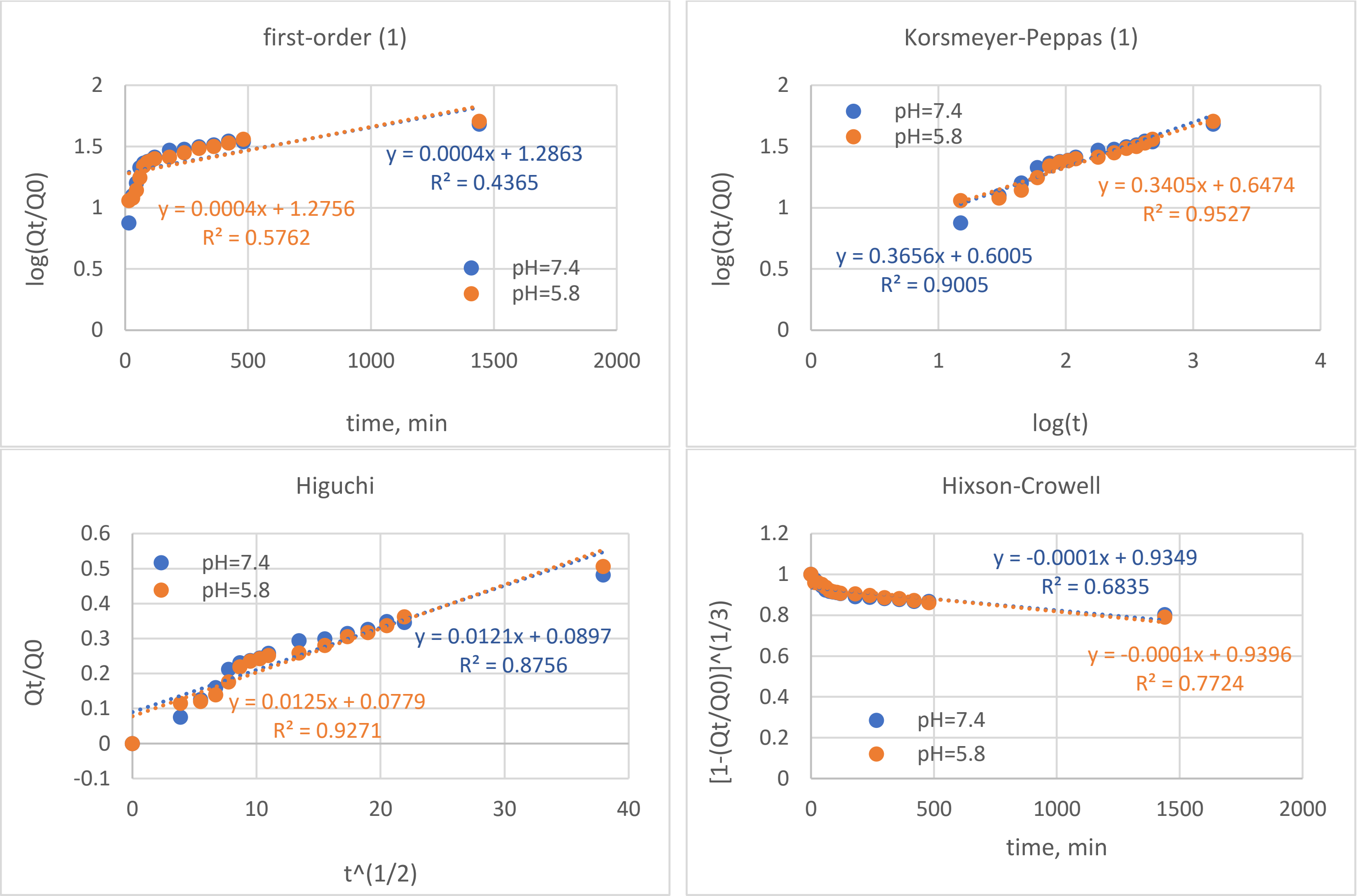

(B) Asiaticoside

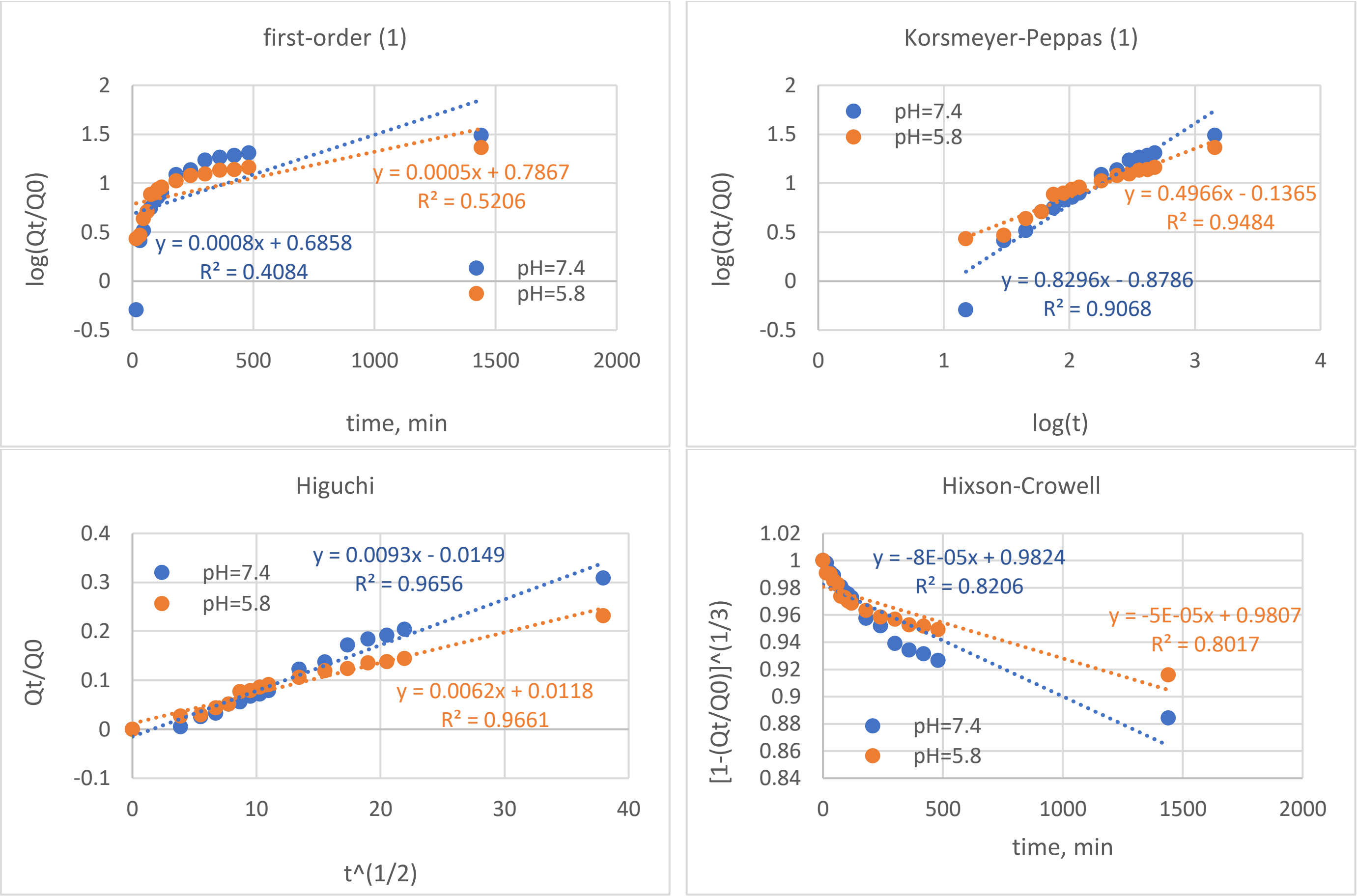

Figure S4. Graphic representation of kinetic models applied to the release profiles from 2 % M+A – W/O sample.

(A) Madecassoside

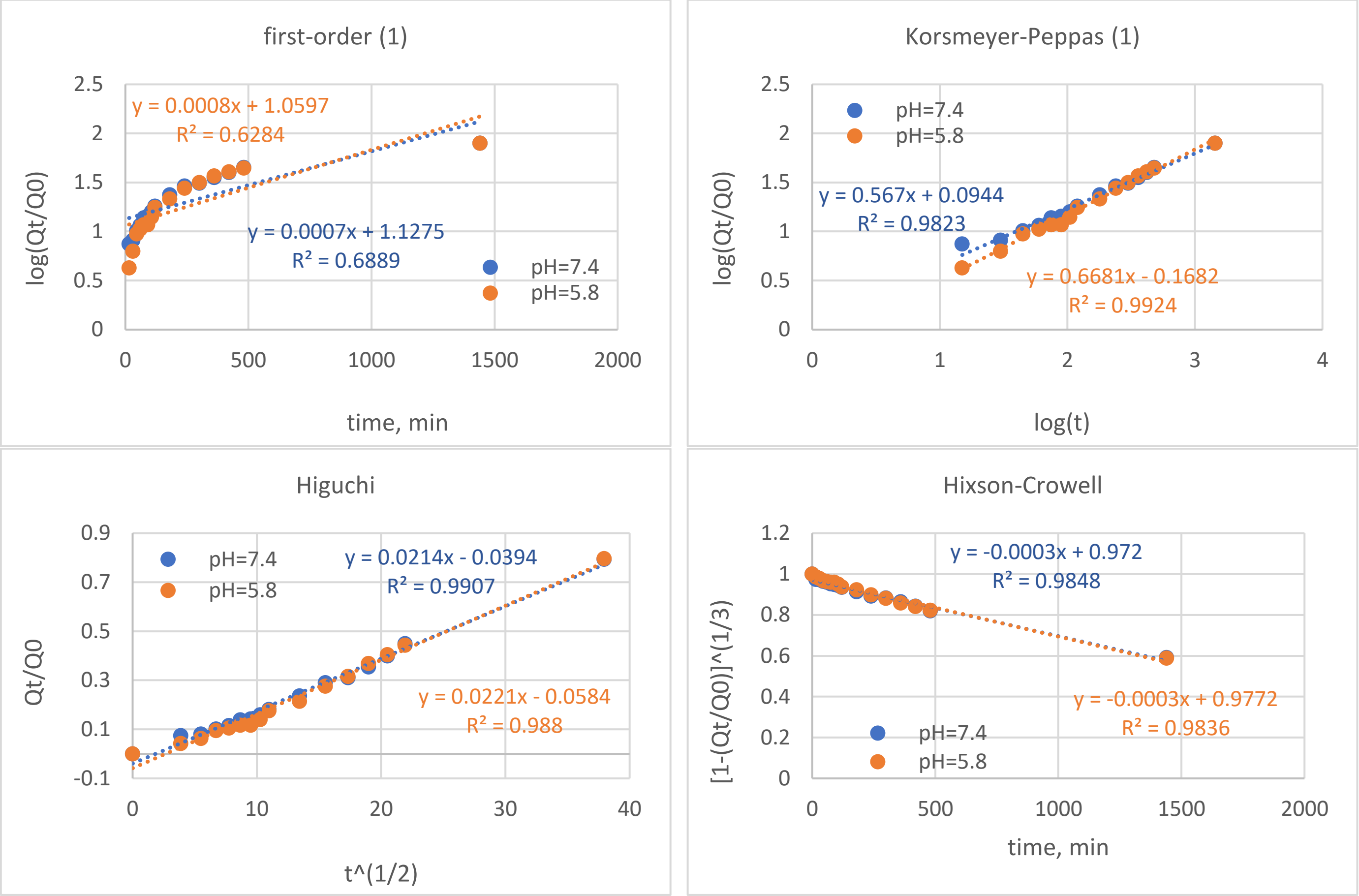

(B) Asiaticoside

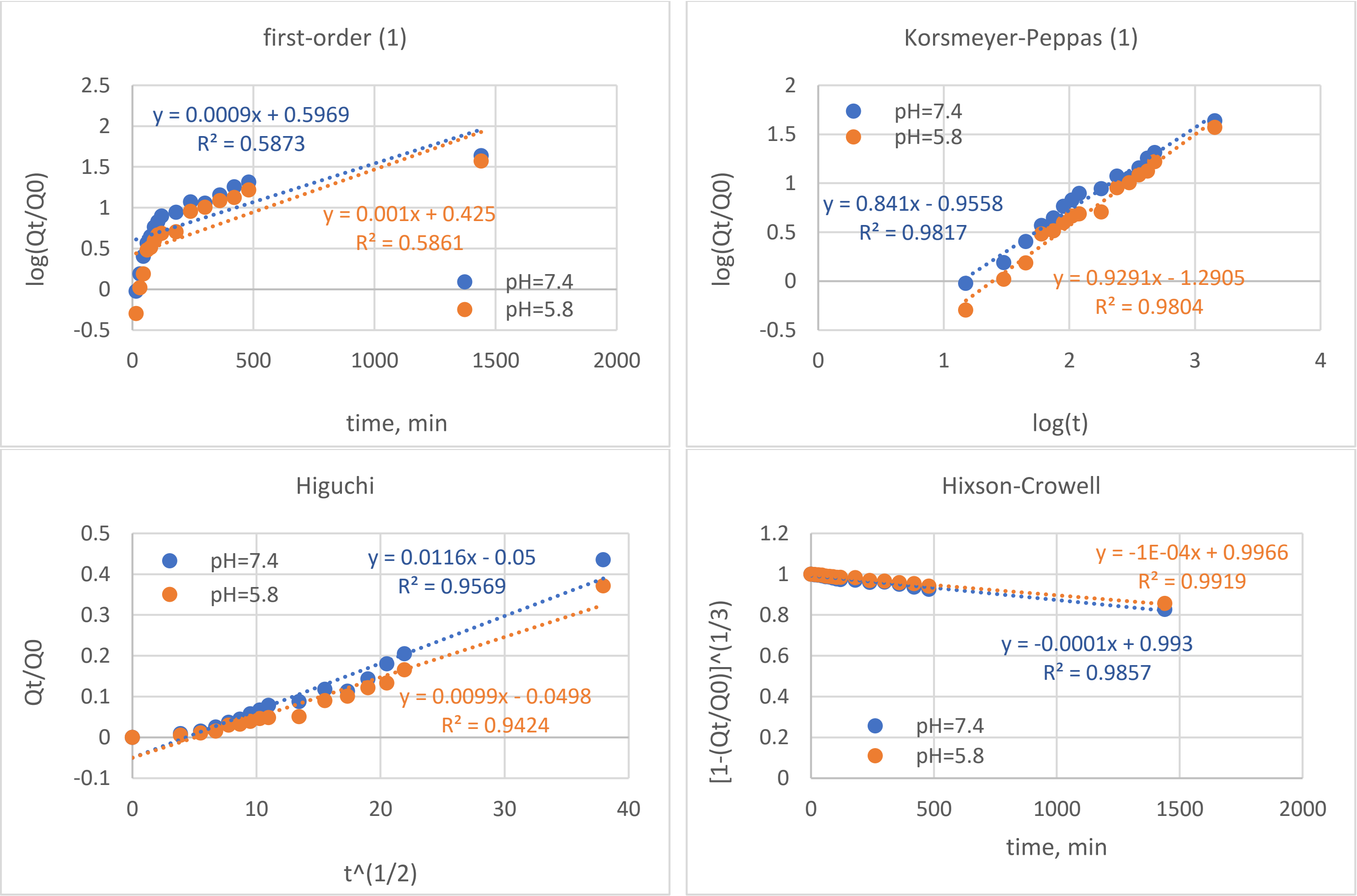

Figure S5. Graphic representation of kinetic models applied to the release profiles from 2 % M+A – G sample.

(A) Madecassoside

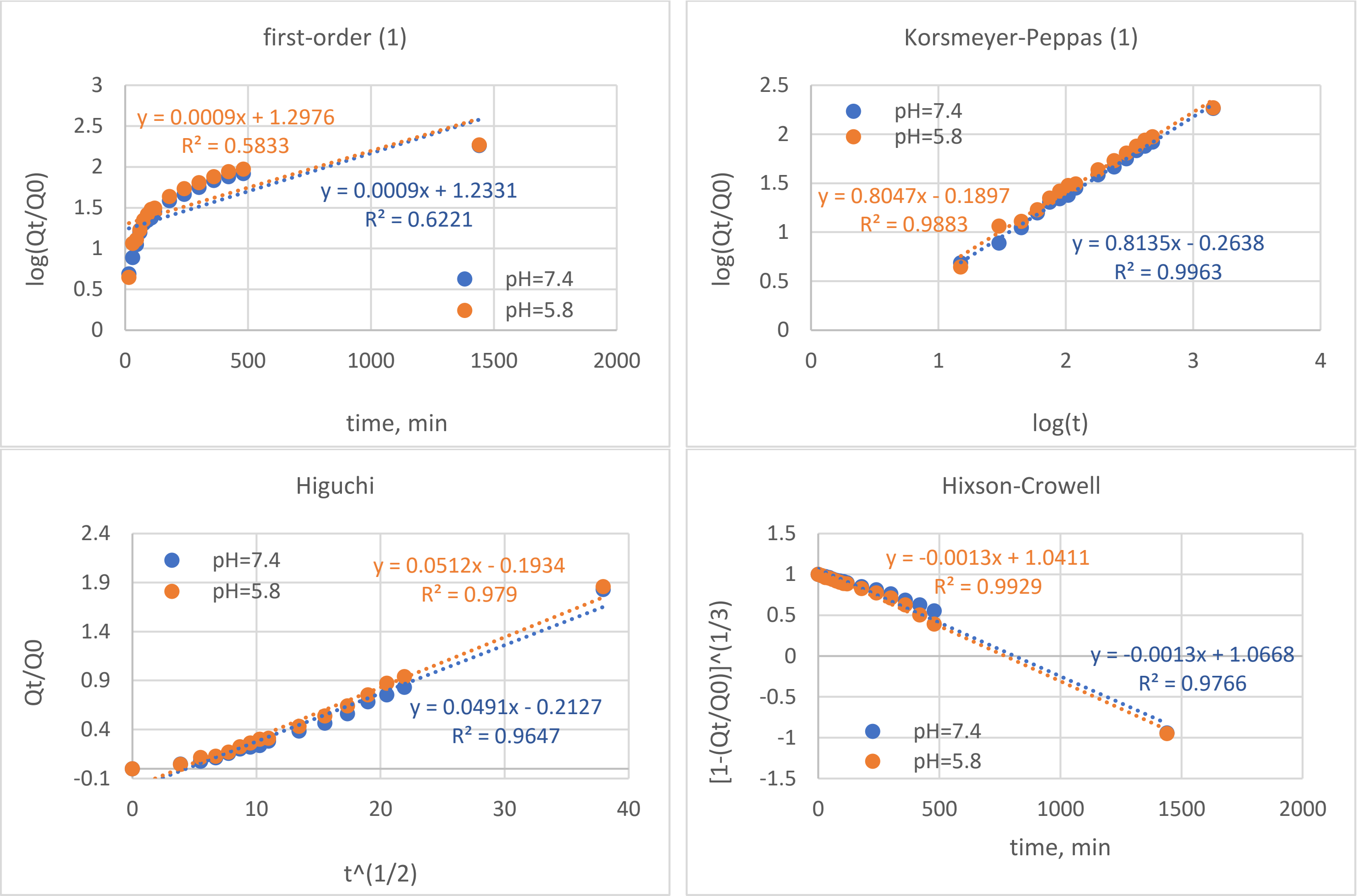

(B) Asiaticoside

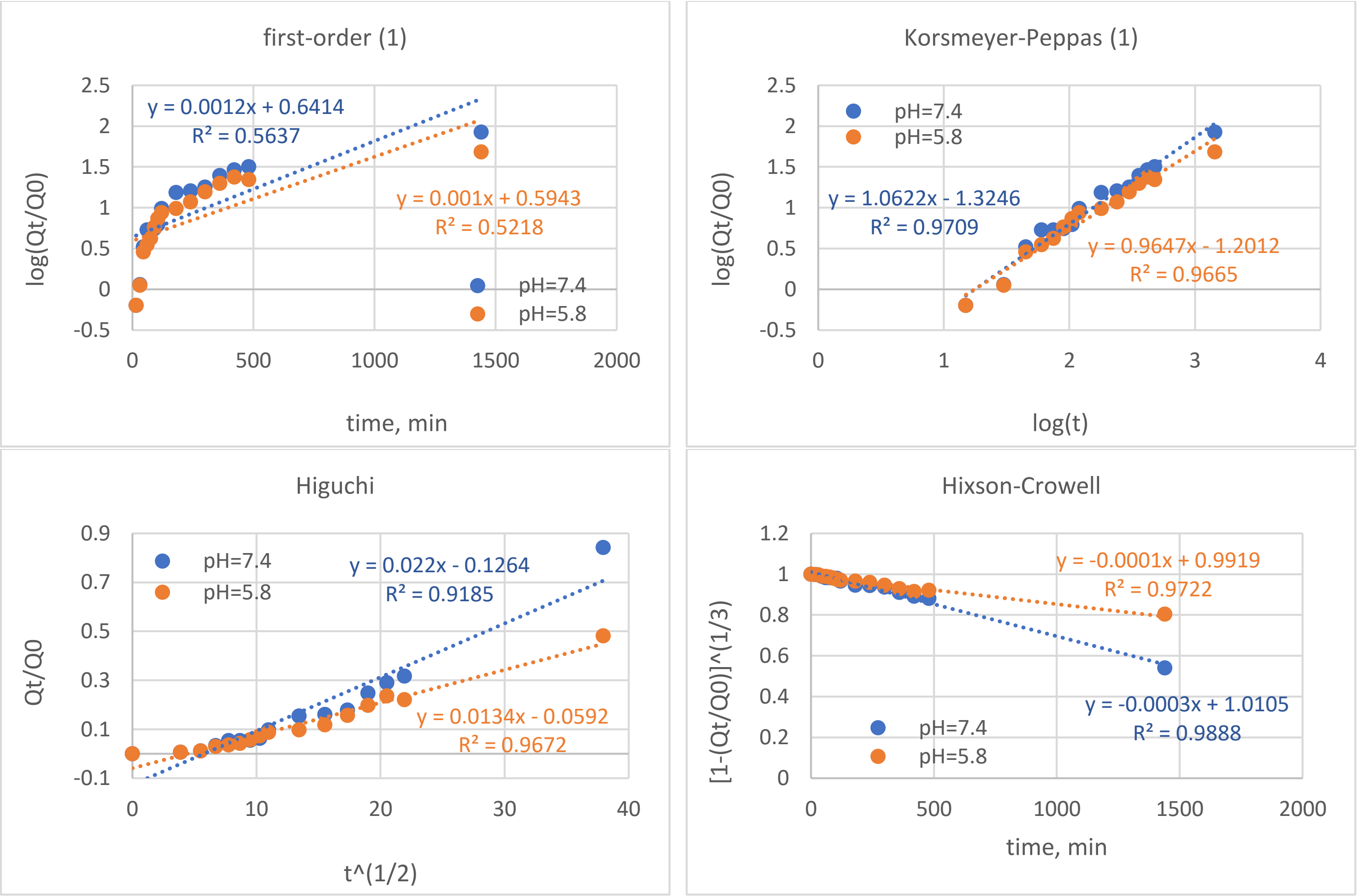

Figure S6. Graphic representation of kinetic models applied to the release profiles from 5 % M+A – O/W sample.

(A) Madecassoside

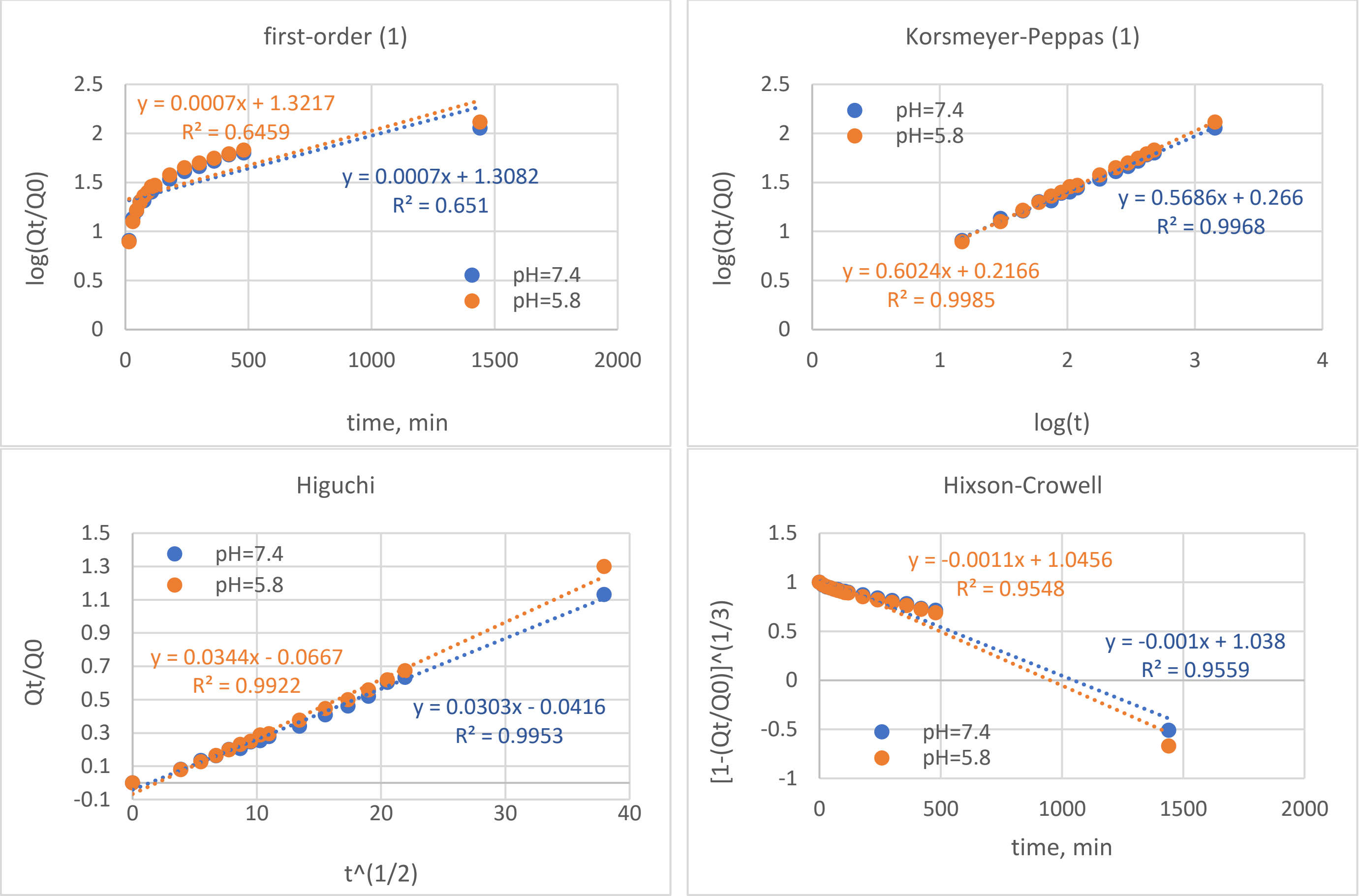

(B) Asiaticoside

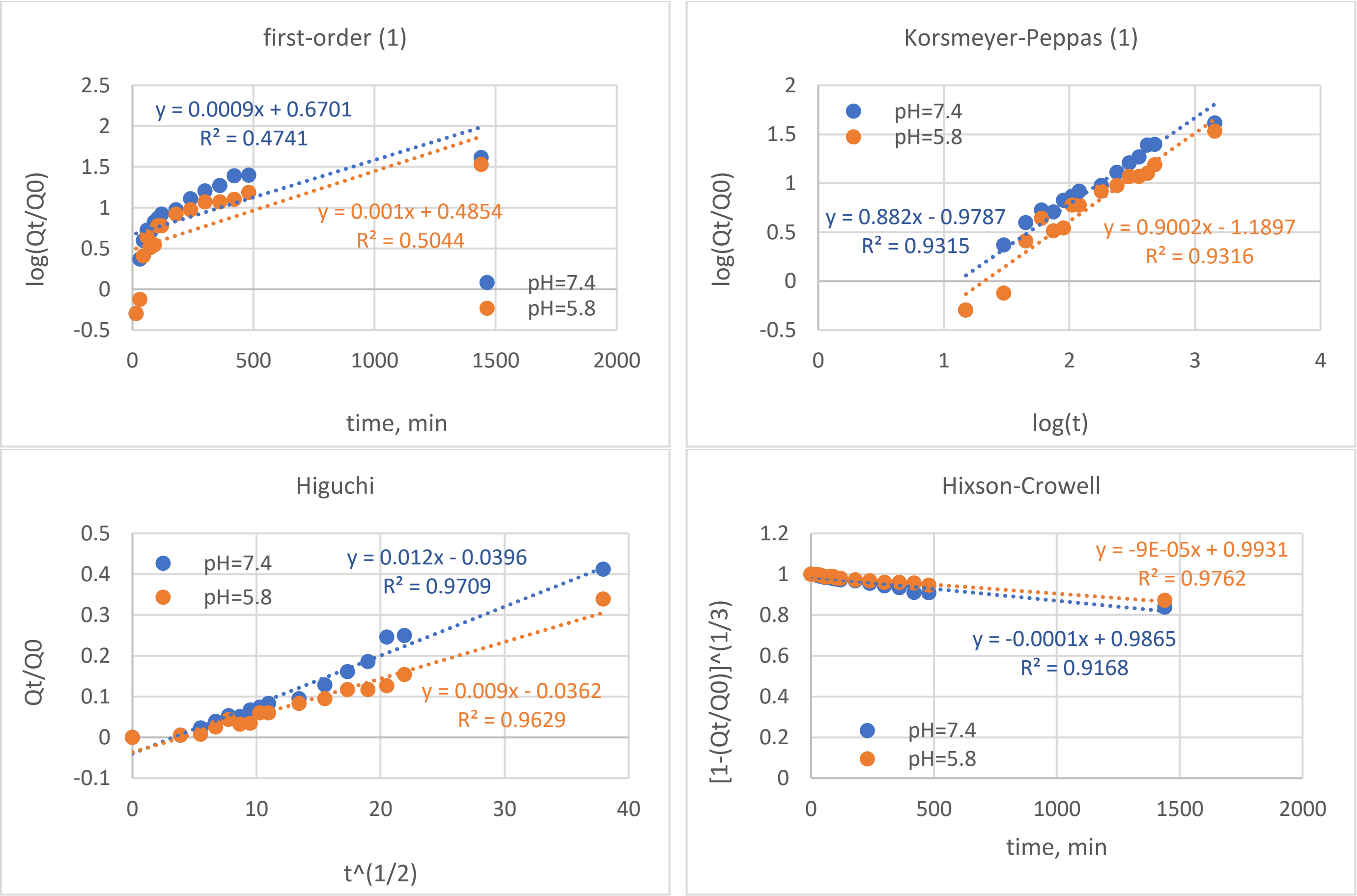

Figure S7. Graphic representation of kinetic models applied to the release profiles from 5 % M+A – W/O sample.

(A) Madecassoside

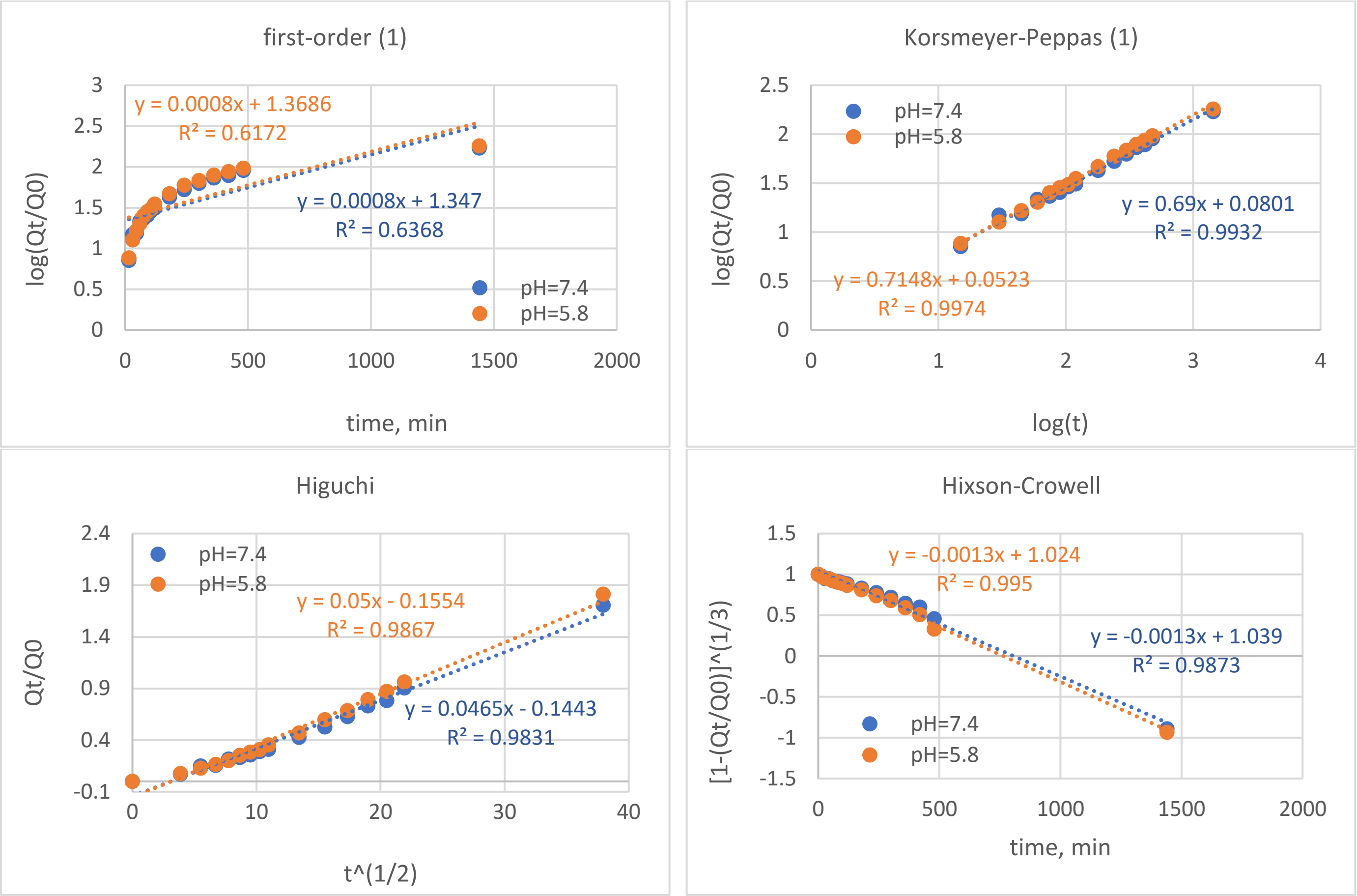

(B) Asiaticoside

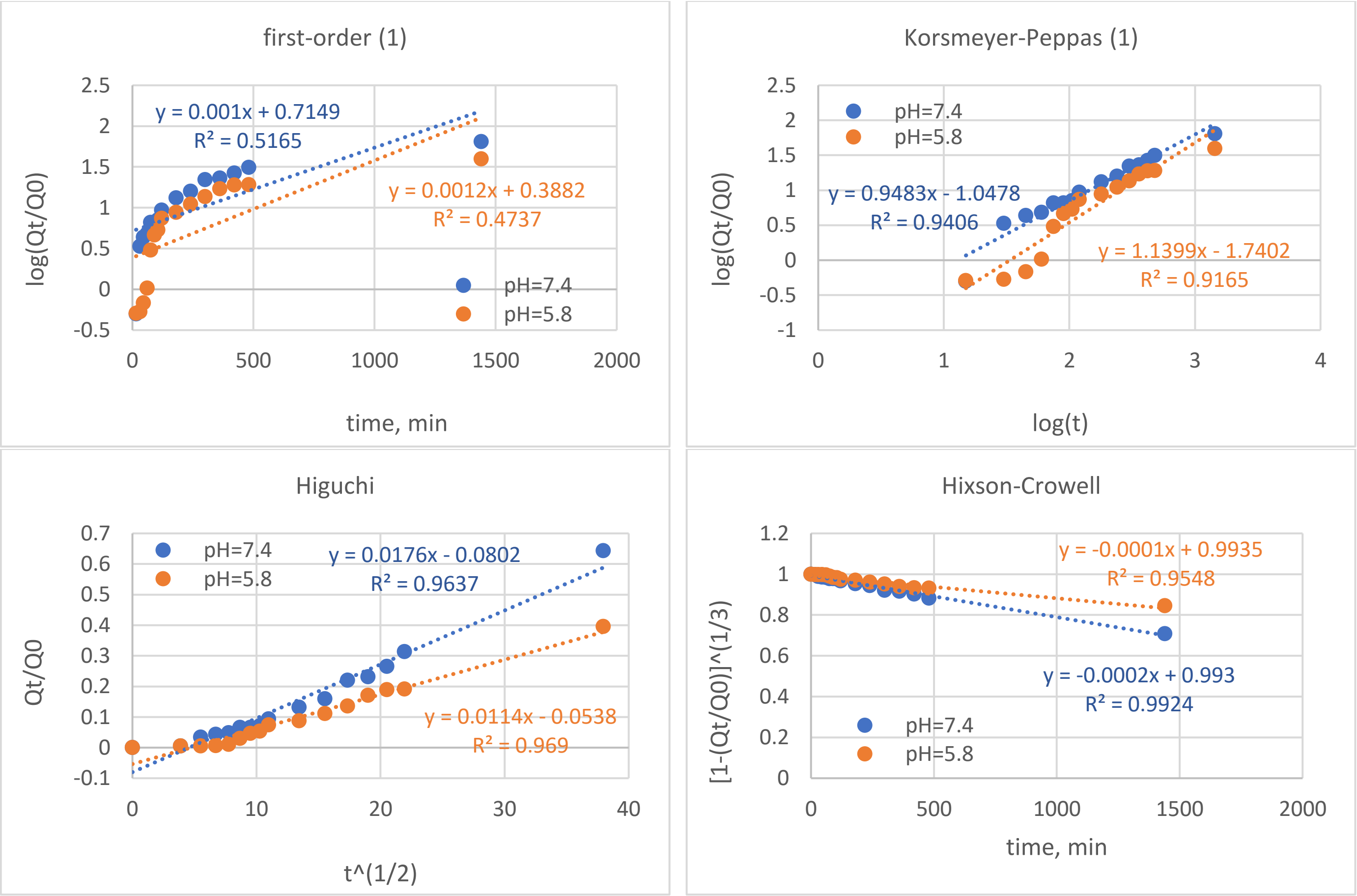

Figure S8. Graphic representation of kinetic models applied to the release profiles from 5 % M+A – G sample.
